# Functional Self-Immolative Hydrogels with Dendritic Cross-Linkers for Controlled Drug Delivery

**DOI:** 10.1021/acs.chemmater.5c01006

**Published:** 2025-07-18

**Authors:** Silvia Muñoz-Sánchez, Jue Gong, Francisco Javier de la Mata, Elizabeth R. Gillies, Sandra García-Gallego

**Affiliations:** † Department of Organic and Inorganic Chemistry and Research Institute in Chemistry ″Andrés M. Del Río″ (IQAR), University of Alcalá, 28805 Madrid, Spain; ‡ Department of Chemistry, The University of Western Ontario, London, Ontario N6A 5B7, Canada; § Networking Research Center on Bioengineering, Biomaterials and Nanomedicine (CIBER-BBN), 28029 Madrid, Spain; ∥ Institute Ramón y Cajal for Health Research (IRYCIS), 28034 Madrid, Spain; ⊥ Department of Chemical and Biochemical Engineering, The University of Western Ontario, London, Ontario N6A 5B9, Canada

## Abstract

In the biomedical field, the design of materials with controlled degradation is highly desired. Herein, we present a family of dendritic hydrogels accomplished through copper-assisted azide–alkyne cycloaddition click reaction between dendritic cross-linkers and complementary linear polymers. As cross-linkers, an innovative family of bifunctional carbosilane dendrimers was designed for this purpose, bearing multiple alkyne groups available for network formation as well as pendant hydroxyl groups for postfunctionalization. Additionally, different azide-pendant polymers were employed, including difunctional poly­(ethylene glycol) with cleavable and noncleavable nature, as well as poly­(ethyl glyoxylate) with and without self-immolative behavior. The rational design of the dendritic hydrogels, through the careful selection of these two components, enabled an accurate manipulation of properties like swelling and mechanical properties. The network degradation could be tuned from a few hours, for a traditional ester-cleavable dendritic hydrogel, to several days under pH-controlled conditions, for the self-immolative hydrogel (SIH). The impact of network degradation on the release of curcumin as a model drug was also confirmed. This work showcased the potential of dendritic SIHs for biomedical applications.

## Graphical abstract

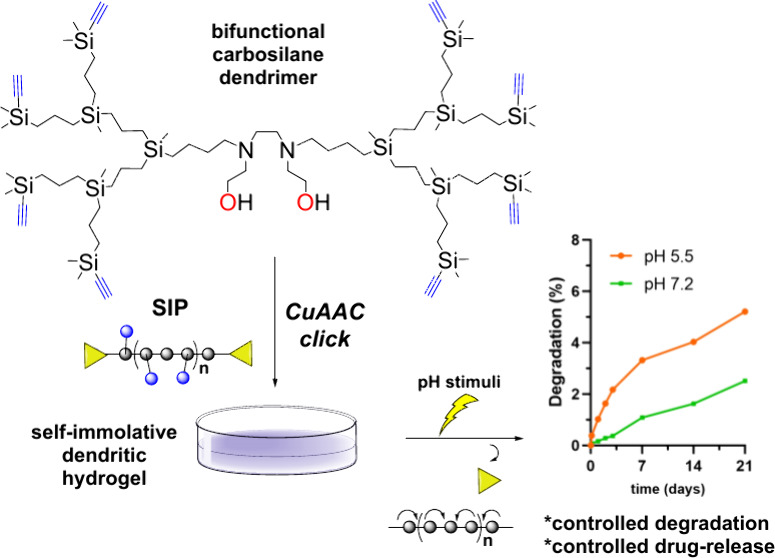

## 1 Introduction

Hydrogels are polymeric networks with a bright future in cutting-edge applications, including drug delivery,^1^ regenerative medicine,^2^ and sensoring.^3^ In these fields, the synthetic control of the hydrogels is highly desired, to enable a precise structure-to-property relationship that can advance the translation to clinical uses. Nevertheless, most of the polymeric hydrogels reported in the literature lack accurate synthetic control.^4^

Dendrimers are highly branched molecules with monodisperse features, offering unprecedented control over the preparation of the resulting dendritic hydrogels.^5,6^ Different dendritic scaffolds have been employed for this purpose, including 2,2-bis­(hydroxymethyl)­propionic acid (bis-MPA) polyesters,^7,8^ poly­(amidoamine)­s (PAMAMs)^9^ and polyglycerols,^10^ among others. In particular, carbosilane dendritic hydrogels have shown promising features due to their amphiphilic nature, which favors interaction with poorly water-soluble drugs.^11^ Additionally, multipurpose networks were easily developed employing bifunctional carbosilane dendrimers,^12^ and stimuli-responsive properties were conferred to the network by carefully selecting the dendritic and polymeric components.^13^

Beyond synthetic precision, accurate control of the hydrogel degradation is also of utmost importance in advanced biomedical applications. Current uses demand the design of smart hydrogels with spatiotemporal control over the degradation sequence. Different parameters, including the nature (physical versus chemical) and density of the cross-linking, the network topology, and other factors affect the degradation profile.^14^ Three main types of degradable hydrogels have been described in the literature: (I) hydrogels that disintegrate along the main chain; (II) hydrogels with cleavable cross-links; and (III) hydrogels with degradable pendant chains. Hydrogels undergo purely bulk degradation, equally throughout the entire network from the surface to the core, as they are hydrated within the networks.^15^ However, the most critical effect is the mechanism of degradation, such as hydrolytic, enzymatic, mechanical, or photoactivated, among others. We have previously shown that carbosilane dendritic hydrogels are quite stable to hydrolytic degradation but can be cleaved in the presence of esterases due to the presence of ester bonds in either the dendrimer core^11^ or in the complementary polymer.^12,13^ This cleavage can also support the release of encapsulated drugs or attached to the network through ester bonds.

Self-immolative polymers (SIPs) are a special type of macromolecules that are programmed to spontaneously disassemble from head to tail in response to stimuli, such as pH change or light.^16^ SIPs are widely recognized as an important class of stimuli-responsive materials for a broad range of applications, such as signal amplification, biosensing, drug delivery, and materials science.^17^ Although early examples included oligomers and dendrimers, many other systems have been developed, including linear polymers, cyclic polymers, graft copolymers, networks, and hyperbranched systems.^18^ The stimuli responsiveness of SIPs and their ability to readily tune their triggering stimulus can provide advantages in hydrogel development. However, very few examples of self-immolative hydrogels (SIHs) have been described, and most of them exhibit poor stability and slow degradation. Gillies and co-workers described the preparation of dendritic SIHs, using self-immolative dendrons with light-responsive moieties at their focal points (Scheme 1a).^19^ The dendrimer generation modulated the hydrogel degradation, achieving slower cleavage with higher generations. To enhance the degradation rate in response to stimuli, they designed SIHs comprising multiarm PEG and self-immolative poly­(ethyl glyoxylate) (PEtG) with a cross-linkable end-cap that responds to light (Scheme 1b).^20^ The hydrogel degradation and drug release could be turned on and off repeatedly through alternating cycles of irradiation and dark storage. More recently, a fully water-soluble SIH was reported that employs strain-promoted azide–alkyne cycloaddition chemistry to potentially allow in situ gelation upon injection.^21^

**Scheme 1:**
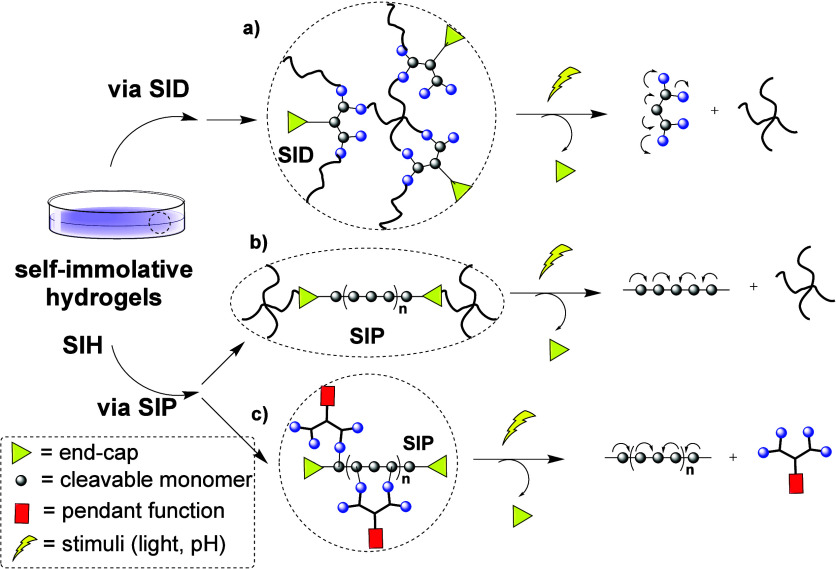
Examples of Self-Immolative Hydrogels, Comprising (a) Self-Immolative Dendrons (SID)^a^, or (b) Self-Immolative Polymers (SIP)^b^; or (c) a Pendant Group^c^

a Adapted from ref.^19^ with permission from the Royal Society of Chemistry.
b Where the end-cap is a crosslinking point, adapted from ref.^20^; Copyright 2023; American Chemical Society.
c As proposed in this work.

In order to improve the control over both the preparation and degradation of the hydrogels, we herein targeted the design of functional dendritic SIHs for drug delivery, combining the advantages of dendrimer cross-linkers and SIPs. A family of bifunctional carbosilane dendrimers was designed to be used as cross-linkers using in situ gelation via CuAAC. Different azide-functional polymers with cleavable, noncleavable and self-immolative behaviors were used for comparison, revealing the outstanding impact of both the dendrimer and the polymer in the structural properties of the hydrogels as well as in the drug loading and release.

## 2 Experimental Section

Comprehensive details of the materials and methods used in this work are described in the Supporting Information. Synthetic protocols for dendritic compounds **D1–D5**, oligomers/polymers **P1–P3** and hydrogels **H1–H5** are described below. The structure and purity of dendritic and polymeric compounds were confirmed via ^1^H, ^13^C, 2D-NMR (Bruker AVANCE Neo400 and 400 MHz Bruker AvIII HD spectrometers); MALDI-TOF (a Bruker Ultraflex TOF/TOF spectrometer); elemental analysis; FT-IR (PerkinElmer FT-IR Spectrum Two instrument) and SEC (Viscotek GPC Max for THF samples, Waters 515 for DMF samples). Hydrogels were characterized by their swelling degree (SD %), gel fraction (GF %), and rheological assays (Discovery Hybrid Rheometer 10 (DHR-10) from TA Instruments (New Castle, DE, USA)). The loading and release of curcumin, as model cargo, from the hydrogels were quantified by HPLC (Agilent 1200).

### 2.1 Synthesis of Alkyne-Functional Dendrons and Dendrimers

#### 2.1.1 Synthesis of **BrGnE_m_** Dendrons

##### 2.1.1.1 General Procedure

A two-step process was used. First, HSiMe_2_Cl was added to a cold solution of precursor BrGnA_m_ in the presence of the Karstedt’s catalyst (one drop) and stirred overnight at 60 °C. Afterward, volatiles were removed under vacuum, ethyl acetate was added, and the solution was filtered through activated carbon under an inert atmosphere. Subsequently, in a second step, BrMg­(CCH) (in ether) was slowly added to the previous solution and the mixture was stirred overnight at room temperature. The solution was washed with brine and dried over MgSO_4_, to give **BrGnE**_**m**_ as a yellowish oil soluble in chloroform (Yield: 76–84%).

##### 2.1.1.2 BrG2E_2_ (**D1**)

It was prepared using the general procedure, using the following reagents: HSiMe_2_Cl (7.67 g, 0.081 mol), BrG1A_2_ (**I**, 5.85 g, 0.027 mol), BrMg­(CCH) (0.0649 mol in 649 mL Et_2_O). C_19_H_37_BrSi_3_ (428.14 g/mol): ^1^H NMR (400 MHz CDCl_3_): 3.32 (2 H, t, *J* = 4.0 Hz, Br-C*H*_2_), 2.30 (2 H, s, CC*H*), 1.74 (2 H, m, Br-CH_2_–C*H*_2_), 1.37 (2 H, m, Br-CH_2_– CH_2_–C*H*_2_), 1.35 (4 H, m, Si-CH_2_C*H*_2_CH_2_-Si), 0.63 (4 H, m, Si-C*H*_2_CH_2_CH_2_-Si), 0.56 (4 H, m, Si-CH_2_CH_2_C*H*_2_-Si), 0.45 (Br-CH_2_–CH_2_–CH_2_–C*H*_2_), 0.09 (12 H, s, Si­(C*H*_3_)_2_), −0.10 (3 H, s, SiC*H*_3_). ^13^C NMR (400 MHz CDCl_3_): δ 92.7 (C*C*H), 87.9 (*C*CH), 35.2 (Br-CH_2_–*C*H_2_), 32.0 (Br-*C*H_2_), 21.2 (Br-CH_2_–CH_2_–*C*H_2_), 19.4 (Si-*C*H_2_CH_2_CH_2_-Si), 17.1 (Si-CH_2_ *C*H_2_CH_2_-Si), 16.9 (Si-CH_2_CH_2_ *C*H_2_-Si), 11.7 (Br-CH_2_–CH_2_–CH_2_–*C*H_2_), −2.9 (Si­(*C*H_3_)_2_), −6.1 (Si­(*C*H)_3_). Elemental analysis: calc. C, 53.11; H, 8.68. Exp.: C, 52.88; H, 9.78. *m*/*z* 428.16; Exp. 429.15 (M+H^+^).

##### 2.1.1.3 BrG3E_4_ (**D2**)

It was prepared using the general procedure, using the following reagents: BrG2A_4_ (**II**, 3.14 g, 6.13 mmol), HSiMe_2_Cl (3.48 g, 36.8 mmol) and BrMg­(CCH) (0.0294 mol). C_41_H_81_BrSi_7_ (848.4 g/mol). ^1^H NMR (400 MHz CDCl_3_): 3.40 (2 H, t, *J* = 4.0 Hz, Br-C*H*_2_), 2.35 (4 H, s, CC*H*), 1.85 (2 H, m, Br-CH_2_–C*H*_2_) 1.41 (2 H, m, Br-CH_2_–CH_2_–C*H*_2_), 1.40 (4 H, m, Si-CH_2_C*H*_2_CH_2_-Si) 1.30 (8 H, m, Si-CH_2_C*H*_2_CH_2_-Si), 0.68 (8 H, m, Si-CH_2_CH_2_C*H*_2_-Si), 0.56 (12 H, m, Si-C*H*_2_CH_2_C*H*_2_-Si-(CH_3_), Si-C*H*_2_CH_2_CH_2_-Si-(CH_3_)_2_), 0.47 (2 H, m, Br-CH_2_–CH_2_– CH_2_–C*H*_2_), 0.15 (24 H, s, Si­(C*H*_3_)_2_), −0.08 (9 H, s, SiC*H*_3_). ^13^C NMR (400 MHz CDCl_3_): δ 93.3 (C*C*H), 89.2 (*C*CH), 36.2 (Br-CH_2_–*C*H_2_), 33.2 (Br-*C*H_2_), 22.3 (Br-CH_2_–CH_2_–*C*H_2_), 20.3 (Si-*C*H_2_CH_2_CH_2_-Si-(CH_3_)_2_), 18.7–18.1 (Si-*C*H_2_ *C*H_2_ *C*H_2_-Si-(CH_3_), (Si-*C*H_2_CH_2_CH_2_-Si-(CH_3_)_2_), 12.7 (Br-CH_2_–CH_2_–CH_2_–*C*H_2_), −2.0 (Si­(*C*H_3_)_2_), −5.2 (Si­(*C*H)_3_). Elemental analysis: calc.: C, 62.49; H, 10.49; Exp.: C, 61.69; H, 10.55; *m*/*z* 848.39; Exp. 849.39 (M+H^+^).

##### 2.1.1.4 BrG4E_8_ (**D3**)

It was prepared using the general procedure, using the following reagents: BrG3A_8_ (**III**, 1.1696 g, 1.15 mmol), HSiMe_2_Cl (1.3 g, 13.8 mmol) and BrMg­(CCH) (0.01104 mol). C_83_H_165_BrSi_15_ (1660.9 g/mol). ^1^H NMR (400 MHz CDCl_3_): 3.39 (2 H, m,Br-C*H*_2_), 2.35 (8 H, s, CC*H*), 1.85 (2 H, m, Br-CH_2_–C*H*_2_), 1.41 (14 H, m, Br-CH_2_–CH_2_–C*H*-CH_2_ Si-CH_2_C*H*_2_CH_2_-Si-(CH_3_) 1.29 (16 H, m, Si-CH_2_C*H*_2_CH_2_-Si­(CH_3_)_2_), 0.68 (16 H, m, Si-CH_2_CH_2_C*H*_2_-Si), 0.57 (40 H, m, Si-C*H*_2_CH_2_C*H*_2_-Si-(CH_3_), Si-C*H*_2_CH_2_CH_2_-Si-(CH_3_)_2_), 0.46 (2 H, m, Br-CH_2_–CH_2_– CH_2_–C*H*_2_), 0.15 (48 H, s, Si­(C*H*_3_)_2_), −0.08 (21 H, s, SiC*H*_3_).

#### 2.1.2 Synthesis of Dendrimers N_2_O_2_GnE_m_

##### 2.1.2.1 General Procedure

The corresponding precursor dendron **D1**–**D3** (2 equiv),^10^ *N,N′*-bis­(2-hydroxyethyl) ethylenediamine (1 equiv), K_2_CO_3_ (3 equiv) and NaI (2 equiv) were added to a stirring flask with the minimum amount of acetone at 90 °C. Once the reaction was complete after 24–72 h, the solution was filtered and the solvent evaporated. The dendrimers were then purified by size exclusion chromatography in acetone. The resulting dendrimers **D4**–**D5** were isolated as yellow oils with a yield of 80–90%.

##### 2.1.2.2 N_2_O_2_G2E_4_ (**D4**)

It was prepared using the general procedure, using the following reagents: Precursor dendron **D1** (3.2612, 8.1203 mmol), *N,N*′**-bis­(2-hydroxyethyl) ethylenediamine (0.602 g, 4.0621 mmol), K_2_CO_3_ (1.6808 g, 12.1619 mmol) and NaI (1.2180 g, 8.1260 mmol). C_44_H_88_N_2_O_2_Si_6_ (844.5 g/mol). ^1^H NMR (400 MHz CDCl_3_): δ 3.58 (4 H, t, *J* = 4.0 Hz, C*H*_2_OH), 2.59 (4 H, m, N-C*H*_2_CH_2_–OH), 2.57 (4 H, s, N-C*H*_2_C*H*_2_-N), 2.49 (4 H, m, N-C*H*_2_CH_2_CH_2_CH_2_-Si), 2.34 (4 H, s, CC*H*) 1.47 (4 H, m, N–CH_2_C*H*_2_CH_2_CH_2_-Si), 1.38–1.27 (28 H, m, N–CH_2_CH_2_C*H*_2_CH_2_-Si, Si-CH_2_C*H*_2_CH_2_-Si-(CH_3_)_2_, Si-CH_2_C*H*_2_CH_2_-Si-(CH_3_)), 0.66 (16 H, m, Si-CH_2_CH_2_C*H*_2_-Si-(CH_3_)_2_), 0.54 (32 H, m, Si-C*H*_2_CH_2_C*H*_2_-Si-(CH_3_)_2_, Si-C*H*_2_CH_2_CH_2_-Si-(CH_3_)), 0.45 (4 H, m, N–CH_2_CH_2_CH_2_C*H*_2_-Si), 0.13 (28 H, s, Si­(C*H*_3_)_2_), −0.08 (9 H, s, SiC*H*_3_). ^13^C NMR (400 MHz CDCl_3_): δ 93.3 (C*C*H), 89.2 (*C*CH), 60.2 (*C*H_2_OH), 55.8 (N-*C*H_2_CH_2_–OH), 55.6 (N-*C*H_2_CH_2_CH_2_CH_2_-Si), 52.7 (N­(*C*H_2_)_2_N), 30.2 (N–CH_2_ *C*H_2_CH_2_CH_2_-Si), 21.7 (N–CH_2_CH_2_ *C*H_2_CH_2_-Si), 20.2 (Si-CH_2_CH_2_ *C*H_2_-Si-(*C*H_3_)_2_), 18.6–18.0 (Si-*C*H_2_ *C*H_2_ *C*H_2_-Si-(CH_3_), Si-*C*H_2_ *C*H_2_CH_2_-Si-(CH_3_)_2_), 13.8 (N–CH_2_CH_2_CH_2_ *C*H_2_-Si), −2.0 (Si­(*C*H_3_)_2_), −5.2 (Si­(*C*H)_3_). Elemental analysis: calc.: C, 57.89; H, 9.60. Exp.: C, 56.80; H, 9.49. *m*/*z* 844.55; Exp. 845.55 (M+H^+^).

##### 2.1.2.3 N_2_O_2_G3E_8_ (**D5**)

It was prepared using the general procedure, using the following reagents: Precursor dendron **D2 (**1.8681 g, 2.1962 mmol), *N,N*′**-bis­(2-hydroxyethyl) ethylenediamine (0.1628 g, 1.0984 mmol), K_2_CO_3_ (0.4553 g, 3.2944 mmol) and NaI (0.3292 g, 2.1963 mmol). C_88_H_176_N_2_O_2_Si_14_ (1749.97 g/mol). ^1^H NMR (400 MHz CDCl_3_): δ 3.58 (4 H, t, *J* = 4.0 Hz, C*H*_2_OH), 2.59 (4 H, m, N-C*H*_2_CH_2_–OH), 2.57 (4 H, s, N-C*H*_2_C*H*_2_-N), 2.49 (4 H, m, N-C*H*_2_CH_2_CH_2_CH_2_-Si), 2.34 (4 H, s, CC*H*) 1.47 (4 H, m, N–CH_2_C*H*_2_CH_2_CH_2_-Si), 1.38–1.27 (28 H, m, N–CH_2_CH_2_C*H*_2_CH_2_-Si, Si-CH_2_C*H*_2_CH_2_-Si-(CH_3_)_2_, Si-CH_2_C*H*_2_CH_2_-Si-(CH_3_)), 0.66 (16 H, m, Si-CH_2_CH_2_C*H*_2_-Si-(CH_3_)_2_), 0.54 (32 H, m, Si-C*H*_2_CH_2_C*H*_2_-Si-(CH_3_)_2_, Si-C*H*_2_CH_2_CH_2_-Si-(CH_3_)), 0.45 (4 H, m, N–CH_2_CH_2_CH_2_C*H*_2_-Si), 0.13 (28 H, s, Si­(C*H*_3_)_2_), −0.08 (9 H, s, SiC*H*_3_). ^13^C NMR (400 MHz CDCl_3_): δ 93.3 (C*C*H), 89.2 (*C*CH), 60.2 (*C*H_2_OH), 55.8 (N-*C*H_2_CH_2_–OH), 55.6 (N-*C*H_2_CH_2_CH_2_CH_2_-Si), 52.7 (N­(*C*H_2_)_2_N), 30.2 (N–CH_2_ *C*H_2_CH_2_CH_2_-Si), 21.7 (N–CH_2_CH_2_ *C*H_2_CH_2_-Si), 20.2 (Si-CH_2_CH_2_ *C*H_2_-Si-(*C*H_3_)_2_), 18.6–18.0 (Si-*C*H_2_ *C*H_2_ *C*H_2_-Si-(CH_3_), Si-*C*H_2_ *C*H_2_CH_2_-Si-(CH_3_)_2_), 13.8 (N–CH_2_CH_2_CH_2_ *C*H_2_-Si), −2.0 (Si­(*C*H_3_)_2_), −5.2 (Si­(*C*H)_3_). Elemental analysis: calc.: C, 62.63; H, 10.51; N, 1.66. Exp.: C, 62.57; H, 10.27; N, 2.415. *m*/*z* 1685.05; Exp. 1687.07 (M+2H^+^).

### 2.2 Synthesis of Azide-Functional PEG Oligomers

#### 2.2.1 Noncleavable Oligomer PEG_400_(N_3_)_2_ (**P1**)

PEG 400 (1 g, 2.5 mmol) and triethylamine (1.39 mL, 10 mmol) were dissolved in DCM under an inert atmosphere and methanesulfonyl chloride (0.77 mL, 10 mmol) was added dropwise at 0 °C. The resulting solution was stirred for 18 h at r.t. and subsequently washed with aqueous NH_4_Cl and then with NaHCO_3_. The organic phase was evaporated to dryness. The mesylate obtained (1.7 mmol, 0.895 g) was dissolved in DMF and sodium azide (3.4 mmol, 221 mg) was added and reacted at 80 °C for 18 h. The volatiles were removed under vacuum and the crude was dissolved in ethyl acetate and washed with aqueous NH_4_Cl. After drying over anhydrous MgSO_4_, the solution was filtered and evaporated to obtain **P1** as a yellowish oil with a yield of 74%.

^1^H NMR (400 MHz CDCl_3_): δ 3.63 (4 H, m, C*H*_2_CH_2_N_3_), 3.60 (28 H, s, OC*H*_2_C*H*_2_O), 3.32 (4 H, t, *J* = 4.0 Hz, CH_2_C*H*_2_N_3_). ^13^C NMR (400 MHz CDCl_3_): 70.6 (O*C*H_2_ *C*H_2_O), 70.0 (*C*H_2_CH_2_N_3_), 50.6 (CH_2_ *C*H_2_N_3_).

#### 2.2.2 Cleavable Oligomer PEG_400_COO­(N_3_)_2_ (**P1d**)

PEG 400 (100 mg, 1 equiv) was dissolved in DCM and 3-azidopropanoic acid (69 mg, 2.4 equiv), 4-dimethylaminopyridine (DMAP, 73 mg, 2.4 equiv) and *N,N*′**-dicyclohexylcarbodiimide (DCC, 124 mg, 2.4 equiv) were added. The solution was stirred for 18 h at r.t. and then filtered several times at 0 °C and washed with aqueous NH_4_Cl. The organic layer was dried over anhydrous MgSO_4_, filtered, and the solvent was removed under vacuum. To eliminate DCU byproducts, the solid was dissolved in water, filtered and evaporated under vacuum to obtain **P1d** as a yellowish oil with 56% yield.

^1^H NMR (400 MHz CDCl_3_): δ 4.17 (4 H, m, COOC*H*_2_CH_2_O), 3.61 (4 H, m, COOCH_2_C*H*_2_O), 3.54 (28 H, s, OC*H*_2_C*H*_2_O), 3.49 (4 H, m, COOC*H*_2_CH_2_N_3_), 2.51 (4 H, m, COOCH_2_C*H*_2_N_3_). ^13^C NMR (400 MHz CDCl_3_): 77.2 (O-(*C*H_2_)_2_-O), 76.0 (COO–CH_2_–*C*H_2_–O), 68.0 (COO–*C*H_2_–CH_2_–O), 54.0 (COO–*C*H_2_–CH_2_–N_3_) 37.8 (COO–CH_2_–*C*H_2_–N_3_).

### 2.3 Synthesis of PEtG and PGAm Polymers

#### 2.3.1 Synthesis of Control Polymers

##### 2.3.1.1 PEtG-M

Freshly distilled EtG (15 mL, 160 mmol, 200 equiv) was placed in a flame-dried Schlenk flask under nitrogen at atmospheric pressure. To this flask, dried *n*-BuLi (372 μL, 0.80 mmol, 1.0 equiv) was added at r.t. and mixed for 10 min, and then dry toluene (50 mL) was added and mixed for 30 min. The solution was then cooled to −20 °C and stirred for 20 min. Dry NEt_3_ (1.67 mL, 12 mmol, 15 equiv) was then added to the polymerization flask and the reaction mixture was stirred for 20 min. Next, dimethylsulfate (2.38 mL, 24 mmol, 30 equiv) was added to the polymerization flask and the mixture was stirred for 1 h at −20 °C, and then placed in the freezer at −15 °C for 48 h. The reaction mixture was precipitated into 500 mL of methanol/water (4/1 v/v). The flask was then sealed and transferred into a–20 °C freezer where it was kept for 5 h. After the liquid was decanted, the precipitate was dried under vacuum to yield an off-white tacky solid. Yield: 48%. ^1^H NMR (CDCl_3_, 400 MHz): 5.66 (br s, 102 H), 4.25 (s, 196 H), 3.52 (s, 3.5 H), 1.32 (s, 302 H), 0.91 (s, 3 H). ^13^C NMR (CDCl_3_, 400 MHz): δ 165.9, 93.2, 62.2, 13.8. FT-IR: 2988, 1745 cm^–1^. SEC (DMF, PMMA): *M*_n_ = 11 000 g/mol, *M*_w_ = 18,000 g/mol, *Đ* = 1.57.

##### 2.3.1.2 PGAm-M (**P2**)

PEtG (1.0 g, 9.80 mmol of ester, 1.0 equiv) was placed into a flame-dried round-bottom flask and stopped with a rubber septum. The flask was evacuated and refilled three times. After the flask was refilled with nitrogen at atmospheric pressure, 20 mL dry 1,4-dioxane was added to dissolve the polymer. The polymer solution was then transferred to a flame-dried Schlenk flask under nitrogen at atmospheric pressure. To this flask, 2-azidoethylamine (473 mg, 5.49 mmol, 0.56 equiv) was added and the reaction was stirred under nitrogen at r.t. The crude reaction mixture was periodically analyzed by ^1^H NMR. The conversion of the pendant ester groups to azide groups was determined by comparing the integration of −CH peak from the polymer backbone at 5.71 ppm with the integration of −CH_2_ peak from the pendant ester groups at 4.25 ppm. The reaction was stopped when ∼30% of pendant ester groups was converted to azide groups. 1,4-dioxane and excess 2-azidoethylamine were removed under vacuum. After the flask was refilled with nitrogen at atmospheric pressure, TEG-amine (7.99 g, 49.0 mmol, 5.0 equiv) was added. The reaction mixture was stirred at 50 °C for 24 h and subsequently dialyzed against deionized water using a 2 kDa MWCO Spectra/Por 6 dialysis membrane (Spectrum Laboratories), and then lyophilized to yield a yellow tacky solid. Yield: 92%. ^1^H NMR (D_2_O, 400 MHz): δ 5.53 (br s, 1.0 H), 3.67–3.48 (m, 8.1 H), 3.36 (s, 1.2 H), 3.30 (s, 2.4 H). ^13^C­{^1^H} NMR (CDCl_3_, 400 MHz): δ 167.2, 96.2, 71.9, 70.4, 69.1, 58.9, 39.3. FT-IR: 3594–3159, 2877, 2103, 1673, 1544 cm^–1^. SEC (DMF, PMMA): *M*_n_ = 14 000 g/mol, *M*_w_ = 22 000 g/mol, *Đ* = 1.59.

#### 2.3.2 Synthesis of Self-Immolative Polymers

##### 2.3.2.1 PEtG-EVE

Freshly distilled EtG (6 mL, 64 mmol, 300 equiv) was placed into a flame-dried Schlenk flask under nitrogen at atmospheric pressure. To this flask, dried *n*-butanol (19 μL, 0.21 mmol, 1.0 equiv) was added and mixed for 10 min at r.t. Then, 24 mL of dry DCM was added at r.t. and the resulting solution was mixed for 30 min. The solution was subsequently cooled to −20 °C and stirred for 20 min. Then dry NEt_3_ (0.18 mL, 1.3 mmol, 6.0 equiv) was added to the polymerization flask and the reaction mixture was stirred for 20 min. Next, ethyl vinyl ether (0.12 mL, 1.3 mmol, 6.0 equiv) was added to the polymerization flask and the mixture was stirred for 5 min at −20 °C, and then TFA (0.20 mL, 2.6 mmol, 12 equiv) was added. The mixture was stirred for 1 h at −20 °C, and then placed in the freezer at −15 °C for 48 h. The reaction mixture was precipitated into 300 mL of methanol/water (4/1 v/v) while adding 0.1 M NaOH to maintain pH ∼ 10. The flask was then sealed and transferred into a −20 °C freezer where it was kept for 5 h. After the liquid was decanted, the precipitate was dried under vacuum to yield an off-white tacky solid. Yield: 30%. ^1^H NMR (CDCl_3_, 400 MHz): 5.66 (br s, 335 H), 5.27 (s, 0.9 H), 4.25 (s, 680 H), 4.05 (s, 2.6 H), 3.79 (s, 2.2 H), 1.31 (s, 1016 H), 0.85 (s, 3.0 H). ^13^C NMR (CDCl_3_, 400 MHz): δ 165.5, 93.2, 62.1, 13.9. FT-IR: 2918, 1750 cm^–1^. SEC (DMF, PMMA): *M*_n_ = 33,000 g/mol, *M*_w_ = 55 000 g/mol, *Đ* = 1.67.

##### 2.3.2.2 PGAm-EVE (**P3**)

This polymer was synthesized by the same procedure as PGAm-M (**P2**). A yellow tacky solid was obtained. Yield: 92%. ^1^H NMR (CDCl_3_, 400 MHz): δ 8.66–7.73 (br s, 0.9 H), 5.71 (br s, 1.0H), 3.76–3.50 (m, 9.4H), 3.43 (s, 1.4H), 3.37 (s, 2.4H). ^13^C­{^1^H} NMR (CDCl_3_, 400 MHz): δ 167.2, 96.6, 71.9, 70.5, 69.1, 58.9, 39.3. FT-IR: 3516–3148, 2870, 2099, 1674, 1545 cm-1. SEC (DMF, PMMA): *M*_n_ = 56 000 g/mol, *M*_w_ = 90 000 g/mol, *Đ* = 1.61.

### 2.4 Synthesis of Dendritic Hydrogels

Hydrogels were prepared from an azide-functional oligomer or polymer (PEG_400_(N_3_)_2_ (**P1**), PEG_400_COO­(N_3_)_2_ (**P1d**), PGAm-M (**P2**) or PGAm-EVE (**P3**)) and a selected dendrimer (N_2_O_2_-G2E_4_ (**D4**) or N_2_O_2_-G3E_8_ (**D5**)) at 15% w/v of both compounds. The reaction was carried out in a mixture of water/THF (1:9), in the presence of CuSO_4_ and sodium ascorbate (NaAsc). For each hydrogel, a different molar ratio was used (Table 1). First, the polymer and the dendrimer were dissolved in THF, mixed and vortexed, and CuSO_4_ and NaAsc were dissolved in water separately. NaAsc was added to the THF solution, it was vortexed, and finally CuSO_4_ was added and it was mixed again. The resulting solution was introduced into several Teflon plugs with a capacity of ∼ 250 μL and gelation occurred overnight at r.t. The hydrogels were then repeatedly washed with an ethylenediaminetetraacetic acid (EDTA) solution until copper was removed.

**Table 1:** Optimized Reaction Conditions and Main Properties of the Dendritic Hydrogels, Including Gel Fraction (GF%), Swelling Degree (SD%), and Crossover Point

**dendrimer**	**polymer**	**hydrogel**	**molar ratio** **[D]:[P]:[Cu]:[Asc]** ^a^	**mass of P (mg)**	**mass of D (mg)**	**GF (%)**	**SD (%)**	**crossover point(% strain)**
N2O2-G2E_4_ (**D4**)	PEG_400_(N_3_)_2_ (**P1**)	**H1**	1:2:0.8:1.6	21.3	22.8	62	89	2.5
N2O2-G3E_8_ (**D5**)	PEG_400_(N_3_)_2_ (**P1**)	**H2**	1:4:1.5:3.0	22.6	24.5	79	47	6.2
	PEG_400_COO(N_3_)_2_ (**P1d**)	**H2d**	1:4:1.5:3.0	23.3	16.1			
	PGAm-M (**P2**)	**H3**	1:1:2.0:4.0	30.3	11.7	81	113	8.0
	PGAm-EVE (**P3**)	**H4**	1:1:2.0:4.0	26.3	10.1	77	55	5.8

a Molar ratio of dendrimer (D), polymer (P), CuSO_4_ (Cu) and sodium ascorbate (Asc). A total polymer and dendrimer concentration of 15% W/V was used.

### 2.5 Studies of Curcumin Loading and Release

#### 2.5.1 Loading Procedure

The selected hydrogel (**H2**, **H3** or **H4**) was immersed in a solution of a curcumin (CUR) solution in ethanol (1 mg/3 mL) for 1 h at 30 °C under light orbital shaking. The gel was then removed from the vial, dried under vacuum, and the remaining solution was analyzed by HPLC to quantify the amount of encapsulated curcumin.

#### 2.5.2 Release Procedure

The CUR-loaded hydrogels **H2** and **H3** were immersed in 3 mL of water and stirred under orbital shaking at 30 °C. CUR-loaded SIH **H4** was immersed in either 3 mL of PBS or sodium citrate buffer and stirred under orbital shaking at 30 °C to evaluate the impact of pH on the self-immolative hydrogel. Samples (50 μL) from the solution were taken over time and CUR was quantified by HPLC.

### 2.6 Degradation of Self-Immolative Hydrogel

SIH **H4** was immersed in 3 mL of either deuterated PBS (pH 7.2–7.6) or deuterated sodium citrate buffer (pH 4.5–5.5) and its degradation over time was monitored by ^1^H NMR spectroscopy.

### 2.7 Postfunctionalization of Dendritic Hydrogels

A glutaraldehyde solution (76 μL, 50% in H_2_O, 5.6 M) was added to a 0.01 M HCl solution in acetone. Subsequently, hydrogel **H2** (22.8 mg) was immersed in the solution and kept with slight stirring (150 rpm) for 20 h. The resultant hydrogel was washed with water and acetone and dried. Rheology assays were used to confirm the success of this reaction.

## 3 Results and Discussion

For the preparation of the dendritic hydrogels, two main components were designed: bifunctional dendrimers decorated with alkyne moieties, used as cross-linkers, and azide-functional polymers with different degradation behaviors.

### 3.1 Synthesis of Bifunctional Carbosilane Dendrimers

Carbosilane dendrimers have silicon–carbon (Si–C) bonds in their structures, which provide excellent kinetic stability, high flexibility, and very low polarity to the macromolecule.^22^ In our previous work,^12^ we designed the first family of bifunctional carbosilane dendrimers, which have a core (*N,N′-*bis­(2-hydroxyethyl)­ethylenediamine, N2O2 for simplicity) available for subsequent functionalization. These bifunctional dendrimers exhibited amphiphilic properties having a polar region (the core) and a nonpolar region of dendritic branches (decorated with vinyl groups). Inspired by these promising materials, we herein designed a family of bifunctional carbosilane dendrimers, in this case with ethynyl groups on the periphery, to pursue different chemistries, such as the highly efficient CuAAC click reaction.

The synthesis of this innovative family of bifunctional dendrimers was carried out through a convergent approach,^12^ detailed in the Section 2. Briefly, as an initial step, the alkyne-functionalized dendrons Br-GnE_m_ were prepared (Scheme 2). Starting from the corresponding allyl-functionalized precursors (Br-G1A_2_ (**I**), Br-G2A_4_ (**II**) and Br-G3A_8_ (**III**)) previously described,^23^ we performed a two-step approach. First, a hydrosilylation reaction with HSiMe_2_Cl in the presence of Karstedt’s catalyst, was performed leading to the air-unstable intermediates (Br-G2Cl_2_, Br-G3Cl_4_ and Br-G4Cl_8_) which were directly taken directly to the second step. Second, these intermediates were reacted with BrMg­(CCH), and after workup, dendrons Br-G2E_2_ (**D1**), Br-G3E_4_ (**D2**) and Br-G4E_8_ (**D3**) were isolated in 80–90% yield. Unlike their vinyl-functionalized counterparts,^12^ dendrons **D1**–**D3** were prepared with a single multiple bond per branch. This change was aimed at increasing the efficiency of the CuAAC reaction, where close triazole groups could generate steric hindrance problems. In fact, no examples in the literature describe the presence of two triazole rings on the same silicon atom.

**Scheme 2:**
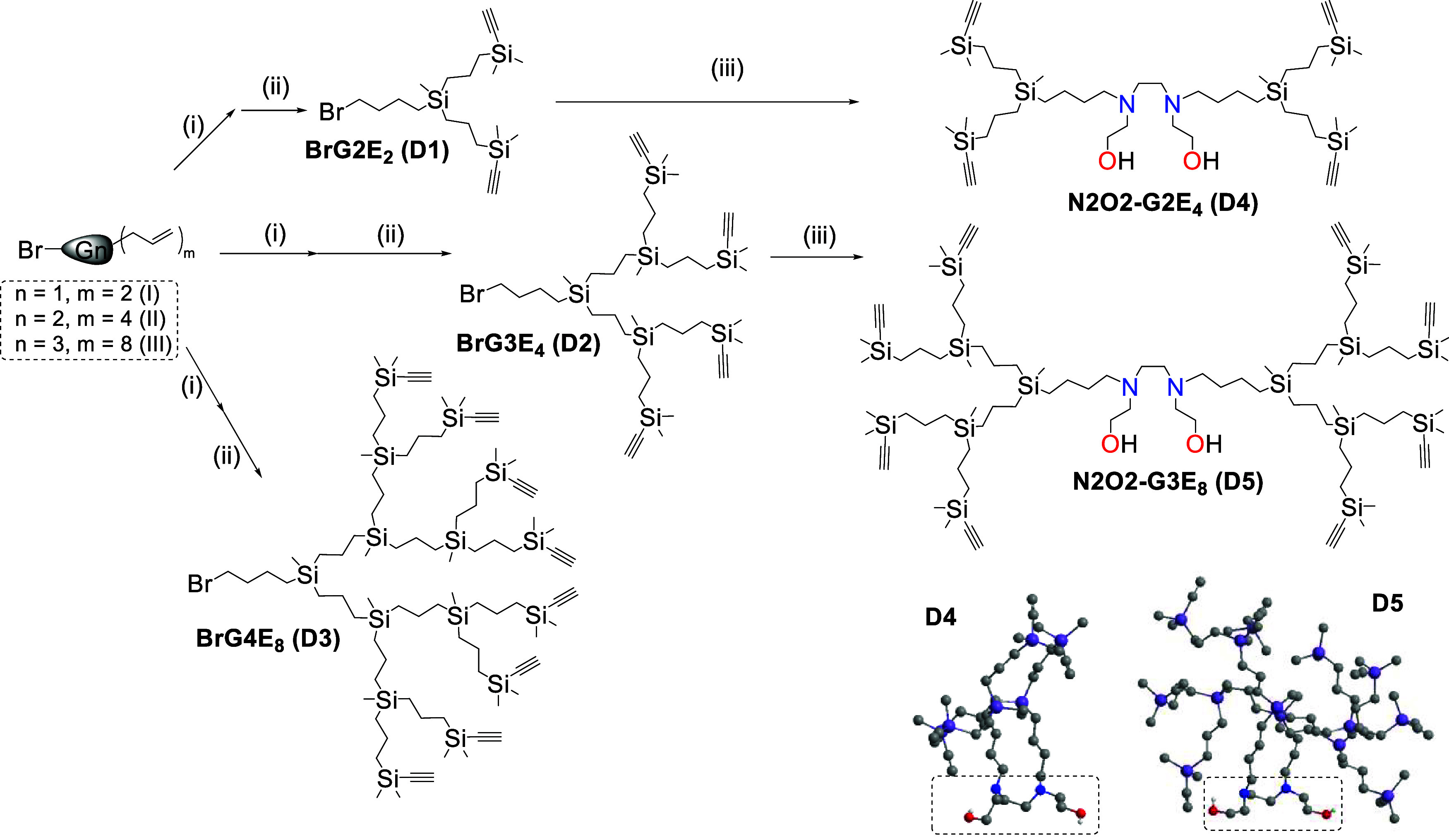
Synthetic Route toward Alkyne-Functional Dendrimers N_2_O_2_-GnE_m_ ^a^

a Reaction conditions: (i) HSiMe_2_Cl (1.5 equiv), Karstedt’s catalyst, 18 h, 60°C. (ii) BrMg­(C_3_H_5_) (1.2 eq./Cl), Et_2_O, 18 h, r.t. (iii) Dendron (2 equiv), *N,N*′**-bis­(2-hydroxyethyl)­ethylenediamine (1 equiv), K_2_CO_3_ (3 equiv) and NaI (2 equiv), acetone, 90°C, 24–72 h. The insets show the snapshots of the 3D spatial arrangement of dendrimers **D4** and **D5** based on molecular dynamics simulations. The dendrimer core is highlighted [Job 1 (Minimize Energy to Minimum RMS Gradient of 0.010) + Job 2 (Molecular Dynamics. Step interval: 2.0 fs. Frame interval: 10 fs. Terminate After: 10000 steps. Heating/Cooling Rate: 1.000 kcal/atom/ps. Target temperature: 300 K)].

For the synthesis of the bifunctional dendrimers, a convergent approach was employed. The corresponding precursor dendron (**D1** or **D2**, 2 equiv) reacted with *N,N*′**-bis­(2-hydroxyethyl)­ethylenediamine (1 equiv), in the presence of K_2_CO_3_ and NaI. The reaction proceeded at 90 °C for 24–72 h. After workup and purification by size exclusion chromatography, the resulting dendrimers N2O2-G2E_4_ (**D4**) and N2O2-G3E_8_ (**D5**) were isolated as yellow oils with 80–90% yield. Unfortunately, the reaction toward the fourth-generation dendrimer did not proceed to completion due to the steric hindrance imposed by the precursor dendron.

The dendritic materials were characterized through ^1^H and ^13^C NMR spectroscopy, using HSQC experiments to assign signals (Figures S1–S13). In ^1^H NMR it was observed that the signal at 3.49 ppm corresponding to Br-CH_2_ of the precursor dendron shifted to 2.59 ppm after binding to the N2O2 core. Unlike the bifunctional vinyl counterparts, which exhibited a characteristic signal between 5.5 and 6.0 ppm assigned to the double bonds, the ethynyl dendrimers presented a characteristic singlet at 2.34 ppm corresponding to the alkyne proton. In ^13^C NMR spectra, it was observed that the signal corresponding to Br-CH_2_ at 33.2 ppm of the precursor dendron disappeared, while a signal appeared around 55.6 ppm corresponding to the N–CH_2_ signal of the dendrimer. Detailed protocols for the synthesis and characterization of all dendritic compounds are summarized in the Section 2 and the Supporting Information.

Furthermore, the purity of our compounds was confirmed by elemental analysis and MALDI-TOF MS. For all dendritic compounds, the molecular peak was identified in mass spectrometry, confirming the presence of the proposed structure (Figures S1–S13). Additionally, molecular dynamics simulations performed with Chem3D v23.1.1. provided information about the 3D conformation of the dendrimers. The N2O2 core is exposed to the environment (Scheme 2, **bottom**), with the branches pointing in other directions. This orientation facilitates their subsequent functionalization, as it will be shown later.

Alkyne-decorated dendrimers are highly interesting materials, as demonstrated with other backbones such as polyester^24^ or PAMAM,^25^ particularly for applications through click chemistry.^26^ Nevertheless, very few examples describe multifunctional alkyne-bearing dendrimers. Malkoch and co-workers described polyester dendrimers that comprise acetylene groups in the interior and hydroxy groups in the periphery. These dendrimers successfully formed hydrogels via CuAAC, with pH tunable degradation (1 h at pH 11 or 4 days at pH 4).^27^

### 3.2 Synthesis of Azide-Pendant Polymers

Two different families of azide-functionalized polymers were prepared. In the first family, PEG 400 g/mol (PEG_400_) was employed as a precursor, as a short linear oligomer with a hydrophilic nature. PEG_400_ was transformed into the noncleavable PEG_400_(N_3_)_2_ (**P1**), through a mesylate intermediate, or into the cleavable PEG_400_COO­(N_3_)_2_ (**P1d**), through esterification with 3-azidopropanoic acid. For both oligomers, the reaction was monitored through ^1^H and ^13^C NMR spectroscopy (Figures S14–S17), confirming the complete conversion of the terminal hydroxyl groups. These oligomers were used as proofs-of-concept to verify the viability of CuAAC as a tool for network formation.

A second family of polymers with azide-pendant moieties was designed (Scheme 3). In this case, poly­(ethyl glyoxylate) (PEtG) was used as the backbone, due to its ability to depolymerize upon end-cap or backbone cleavage. Polymerization of purified ethyl glyoxylate (EtG) was initiated with *n*-butanol in the presence of NEt_3_ and was performed at −20 °C due to the polymer’s low ceiling temperature. TFA and ethyl vinyl ether (EVE) were added to end-cap the polymer, because the resulting acetal end-cap should lead to pH-sensitive cleavage and consequently depolymerization. The polymer had *M*_n_ of 56,000 g/mol and dispersity (*Đ*) of 1.61. PEtG-EVE was then reacted with 2-azidoethylamine in dry dioxane at r.t., until 30% amidation was accomplished as monitored by ^1^H NMR spectroscopy. Subsequently, the dioxane was evaporated and the product reacted with TEG-amine at 50 °C for 24 h to introduce hydrophilic groups. SIP polymer **P3** was dialyzed and lyophilized until a tacky solid was obtained with 92% yield. A similar protocol was followed to prepare the control polymer **P2**, where the EVE end-cap was substituted with a methyl group, and the self-immolative properties disappeared. These polymers were also fully characterized through NMR, FT-IR and SEC (Figures S18–S27).

**Scheme 3:**
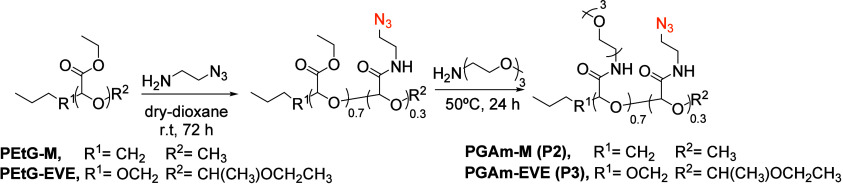
Synthesis of the PGAm Polymers **P2** (PGAm-M, Control) and P3 (PGAm-EVE, Self-Immolative Polymer) with Multiple Azide Pendant Groups

### 3.3 Synthesis of Dendritic Hydrogels

The hydrogels were synthesized through CuAAc click reaction, Figure 1. The ethynyl-functionalized dendrimers were cross-linked with the four different polymers previously described: the PEG-based oligomers **P1** and **P1d**, and the PGAm polymers PGAm-M (**P2**) and PGAm-EVE (**P3**), the latter with self-immolative activity. A thorough study was performed to optimize the reaction conditions, switching the reagent ratios, the concentration, and the solvent. Table 1 summarizes the results of the optimized conditions for each hydrogel. In general, the reactions were carried out in a mixture of water/THF (1:9), using 15% (w/v) of dendrimers and polymers with different molar ratios. CuSO_4_ and sodium ascorbate were used to generate Cu­(I) *in situ*. The resulting mixture was introduced into Teflon plugs with a capacity of around 250 μL and reacted for 18 h under orbital shaking. The hydrogels were then washed several times with an EDTA solution until the remaining catalyst was removed. A satisfactory elimination was confirmed through ICP-MS, which revealed copper values below 0.2 μg/mg in the tested hydrogels.

**Figure 1:**
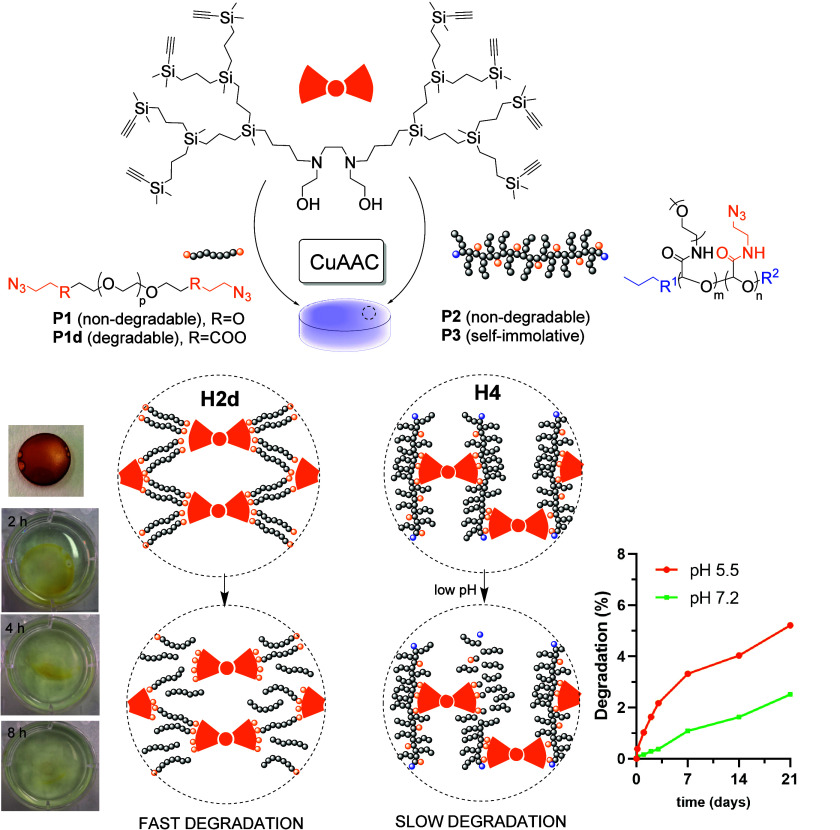
General synthetic scheme toward carbosilane dendritic hydrogels generated through CuAAC, using dendrimer N2O2-G3E_8_ (**D5**) as cross-linker and an azide-functional polymer. Left: PEG-based oligomers (**P1** and **P1d**), showing the fast degradation of **H2d** upon exposure to water. Right: PGAm polymers (**P2** and **P3**), showing the controlled degradation of the self-immolative hydrogel **H4**.

### 3.4 Cross-Linking and Swelling Studies

The efficiency of the cross-linking reaction is represented by the gel fraction (GF%, Table 1). These hydrogels, obtained through CuAAC, present GF% in the range of 60–80%, being higher for the third-generation dendrimer **D5**. This trend can be attributed to the higher number of cross-linking points that form the network, resulting in a more stable network. Surprisingly, very similar GF% was obtained for all the hydrogels prepared from **D5**. This may indicate that the limitations are mainly imposed by the steric hindrance and conformation of the dendrimers. This result also confirms the robustness of click reactions in the preparation of these hydrogels, but with lower efficiency for CuAAC (60–80%) than for the thiol–ene coupling (80–90%).^12^ The high efficiency of the cross-linking reaction was also confirmed through FT-IR analysis. In the precursor polymers, the azide group shows a sharp peak at ∼2100 cm^–1^, while the terminal alkyne groups from the dendrimers appear as two sharp peaks at 2030 cm^–1^ (C≡C) and at ∼3300 cm^–1^ (≡C–H). After CuAAC reaction, these bands seem to disappear or decrease significantly (Figures S28 and S29).

To evaluate the properties and potential uses of these dendritic hydrogels, we analyzed the swelling degree (Figure 2). For **H1** and **H2**, both prepared from the same oligomer **P1** but using different generation cross-linkers, the SD% follows the same trend as in previous works.^12^ The increase in dendrimer generation led to a more lipophilic network, and thus SD% substantially decreased from 89% in **H1** to 47% in **H2**. The size of this type of polymer also influences the swelling of the hydrogel. The PEG_400_ used in this work is shorter than in the previous work (PEG_1k_) and generates hydrogels with lower swelling. The nature of the polymer is also crucial. The main difference between **H2** and **H2d** is the presence of cleavable ester bonds in the latter. Although **H2** showed high stability during the span of the experiment (48 h), the fast degradation observed in **H2d** hindered the evaluation of the SD% for this hydrogel (Figure 1, left).

**Figure 2:**
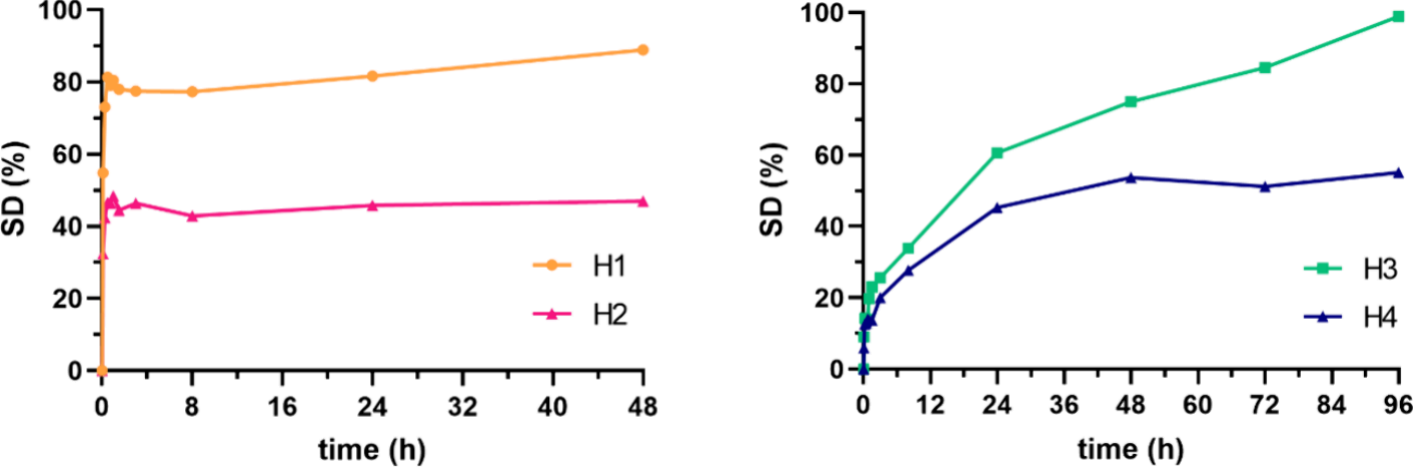
Swelling degree (%) of hydrogels **H1** and **H2** (prepared from oligomer **P1**, left), and hydrogels **H3** and **H4** (prepared from polymers **P2** and **P3**, right).

Figure 2 (right) shows the SD% of **H3** and **H4** hydrogels. These hydrogels were prepared with dendrimer N2O2-G3E_8_ (**D5**) and polymers PGAm-M (**P2**) or PGAm-EVE (**P3**). Unlike hydrogels prepared with PEG_400_ (**P1**), which show a very fast swelling and then stabilize, these show a more gradual swelling probably related to the longer nature of the PGAm polymer. Hydrogel **H3**, which was prepared with a shorter polymer, has a SD of 113% compared to 55% for **H4**, which is prepared with a longer polymer, suggesting that the hydrogel prepared from the longer polymer may have provided a more stable, homogeneously cross-linked network. Thus, overall the swelling arises from a combination of factors such as the hydrophilicity of the materials, length of the polymers, and homogeneity of the polymer network.

### 3.5 Mechanical Characterization of Dendritic Hydrogels

The study of the mechanical properties of hydrogels is essential to understand the potential applications in different fields, such as biomedicine, tissue engineering, and controlled drug release. Hydrogels exhibit viscoelastic properties, meaning they have characteristics of both viscous liquids and elastic solids. The storage or elastic modulus (*G*′) measures the amount of elastic energy stored in the material during deformation, and the loss or viscous modulus (*G*″) measures the amount of energy dissipated as heat during deformation. A high *G*′ indicates a more solid behavior, while a high *G*″ indicates a more liquid behavior. We obtained these parameters through rheology under oscillatory experiments.

#### 3.5.1 Impact of the Dendrimer and the Polymer on the Mechanical Properties

Initially, we performed an amplitude sweep test in the range of 0.1–100% at constant frequency (1 Hz). This experiment identified the linear viscoelastic region (LVR), where deformation does not lead to irreversible changes. During the test, the response of the material was measured in terms of the storage modulus (*G*′), loss modulus (*G*″) and the crossover point (*G*′ = *G*″). This critical point provides information about the cross-linking density: A crossover point that occurs at a low strain amplitude indicates a weaker or less densely cross-linked network. As summarized in Table 1, all hydrogels have a critical point below 10% strain, following the trend **H1** (2.5%) < **H4** (5.8%) < **H2** (6.2%) < **H3** (8%). These data precisely correlate with GF%, suggesting a more flexible and less dense internal structure for **H1**.

Subsequently, frequency sweep tests were performed at constant strain (1%) and increasing frequency (0.1–10 Hz), to evaluate how the hydrogels respond to different deformation frequencies. The difference between the storage modulus (*G′*) and the loss modulus (*G*″) as a function of frequency is crucial to understanding the mechanical and dynamic properties of the material. When *G′* ≫ *G* *″* the material behaves primarily as an elastic solid and when *G′* ≈ *G*″, it means that the material exhibits balanced viscoelastic behavior, in which the elastic and viscous properties of the material are comparable. As shown in Figure 3, hydrogels **H1** and **H2**, which were prepared from the same oligomer **P1** but different generation dendrimers, have a similar pattern. In both cases, *G′* and *G*″ remain relatively constant in the range of frequencies. However, we can see a significant difference between *G* *′* and *G*″. **H1** has a balanced behavior between the elastic and viscous moduli (*G′* ≈ *G*″), whereas **H2** has a predominantly elastic behavior (*G* *′* ≫ *G*″). **H3** and **H4**, which are prepared from PGAm-M (**P2**) and PGAm-EVE (**P3**) respectively, exhibit a totally different pattern. Both show a balanced behavior between the two moduli at low frequencies; however, as the frequency increases, the difference between the two moduli increases considerably with a predominance of elastic behavior. The different length of the polymer chains clearly affects this behavior. PGAm-EVE, which has a 3-fold higher *M*_n_ than PGAm-M, is more prone to chain entanglements and this reduces the elasticity of the hydrogel. For all hydrogels, the mechanical properties pattern resembles the SD% curves, suggesting a connection between both properties.

**Figure 3:**
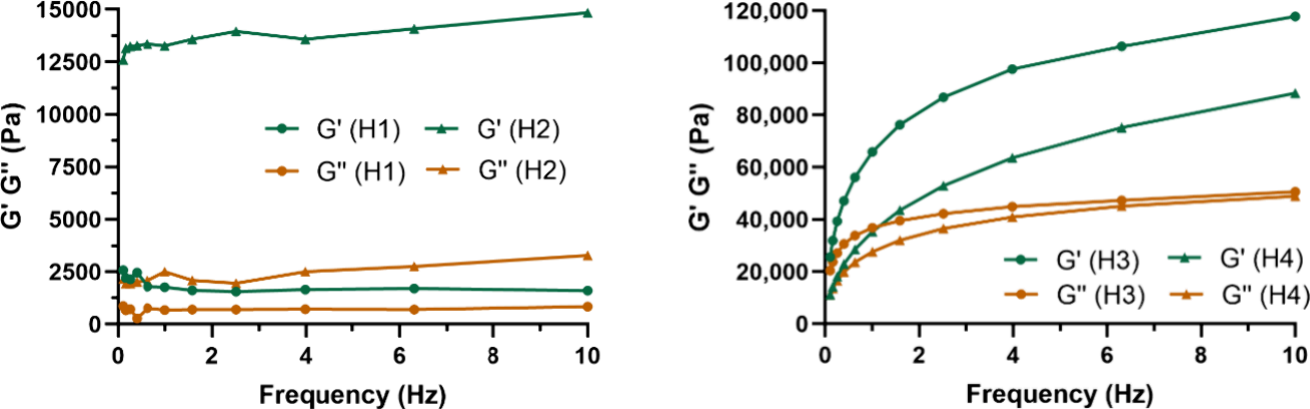
Comparative frequency sweep assays for **H1** and **H2** (left), **H3** and **H4** (right).

#### 3.5.2 Modulation of the Mechanical Properties through Dynamic Covalent Bond Formation

The bifunctional nature of the dendritic cross-linkers **D4** and **D5** reported herein is highly interesting in materials chemistry. While the multiple alkyne groups can be used to cross-link the network, the presence of pendant hydroxyl groups on the dendrimers core offers additional advantages. For example, they can be used for the attachment of bioactive molecules and allow controlled release under certain stimuli.^12^ Nevertheless, we employed these additional groups to fine-tune the mechanical properties of the hydrogels. As a proof-of-concept, hydrogel **H2** was reacted with a glutaraldehyde solution under acid conditions for 18 h, with gentle stirring. After isolation, the hydrogel **H2-Glu** was studied using amplitude and frequency OSR assays. The reaction led to reversible acetal bonds that linked the dendrimer cores, reinforcing the cross-linking of the hydrogel (Figure 4). The critical point of the hydrogel increased from 6.2% in **H2** to 12% in **H2-Glu**, suggesting a higher degree of cross-linking and a denser and more rigid internal structure.

**Figure 4:**
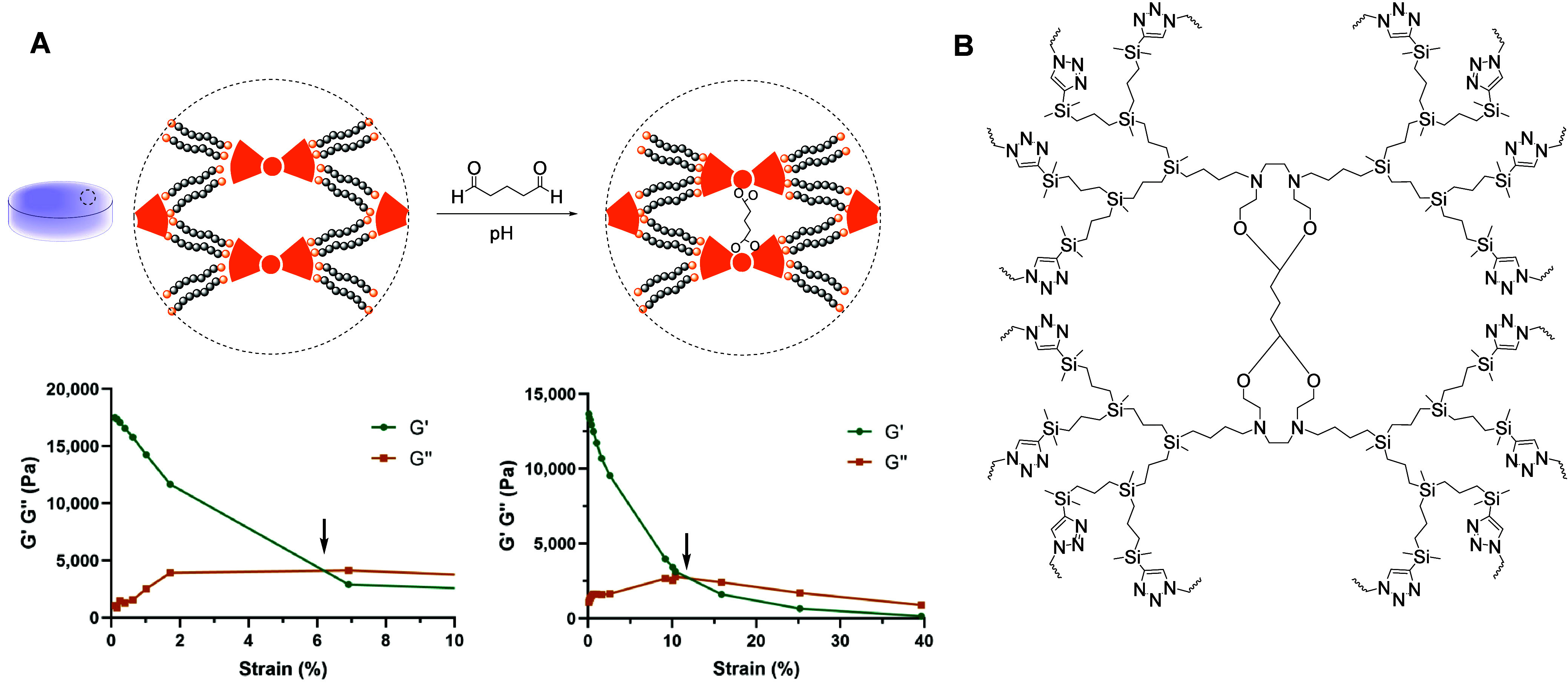
(A) Change of mechanical properties from **H2** to acetal-reinforced hydrogel **H2-Glu**, highlighting the critical point (arrow). (B) Expanded structure of the intercore cross-linking.

### 3.6 Exploring the Degradation Profile of the Self-Immolative Hydrogel through NMR Spectroscopy and Rheology

Hydrogel **H4** presents multiple self-immolative polymer (**P3**) chains in its structure. This polymer can undergo a pH-dependent cleavage of the end-cap (EVE), triggering the sequential release of the different monomeric units. To evaluate the degradability of **H4** in response to pH, the SIH was immersed in 1 mL of either deuterated PBS (pH 7.2–7.6) containing acetone as an internal standard for quantification, or deuterated sodium citrate buffer (pH 5.5). The hydrogel degradation was monitored by ^1^H NMR spectroscopy in D_2_O (Figures 5 and S30). The cleavage of the end-cap led to network degradation and the release of compounds A and B, as evidenced by the appearance of characteristic peaks at δ 5.29 (a), 3.46 (f), 3.38 (d), 0.16 (g) and 0.12 (h) ppm. The degradation rate was quantified from the integral corresponding to −C*H*(OH)_2_ (“a” proton) at 5.29 ppm, compared to the standard peak of acetone (2.25 ppm) for the pH 7.2 assay or to the standard peak of citrate buffer (0.96 ppm) for the pH 5.5 assay. Note that these peaks are only observed when molecules A and B are released to the media. Even if the network starts degrading soon, the release of molecule B–comprising eight cross-linking points- would require more time to be observed. This experiment confirmed that the depolymerization was around three times faster at pH 5.5 (Figure 1, right).

**Figure 5:**
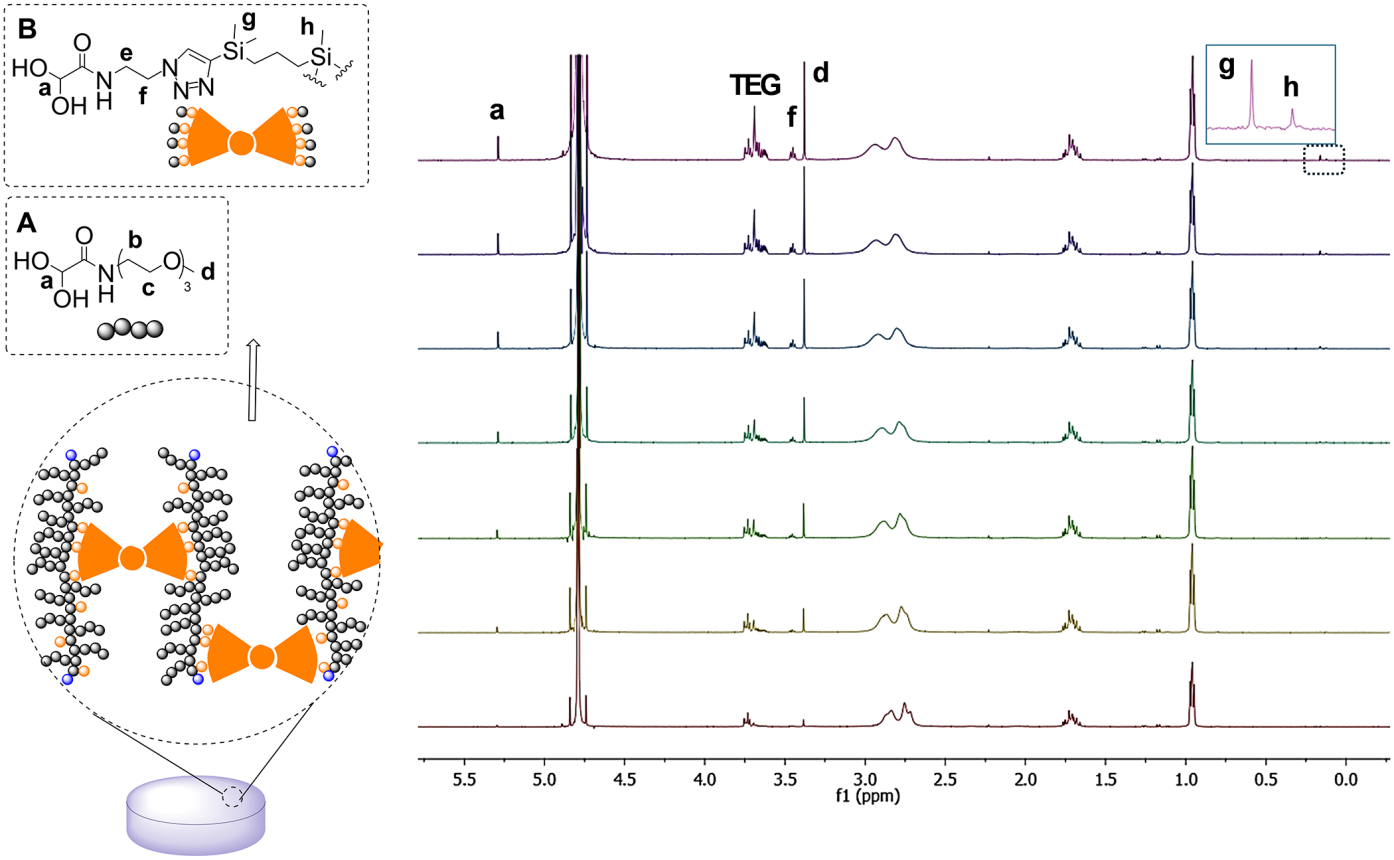
Degradation study of the self-immolative hydrogel **H4** at pH 5.5, as monitored through ^**1**^H NMR in D_2_O, highlighting selected signals of the released compounds A and B over time (from bottom to top: degradation products at 3, 24, 48, 72 h, then 7, 14, and 21 days. Insert: Zoom in on spectra to identify dendrimer signals.

To confirm the real impact of the degradation, rheology was used to evaluate the change in storage modulus (*G′*) over time at pH 5.5. Frequency sweeps revealed a fast decrease in the value of *G* *′*, indicating that the hydrogel rapidly loses its structural integrity (Figure S31).

### 3.7 Drug Loading and Release Studies

Many common drugs have poor water solubility, which significantly affects their bioavailability, dosing frequency, and patient compliance. Hydrogels can encapsulate drugs and release them in a sustained manner over time, mitigating these issues. In our previous work,^12^ we were able to load a poorly water-soluble antibiotic drug, such as ciprofloxacin, thanks to the amphiphilic nature of carbosilane dendritic hydrogels which improved the compatibility with drugs with very low polarity. In this work, we selected curcumin (CUR), a potent anti-inflammatory drug that is also very poorly water-soluble (0.6 μg/mL), as proof-of-concept. Hydrogels **H2**, **H3** and **H4** were selected due to similar GF% but different internal structure. Hydrogels were immersed in CUR ethanol solution (1 mg/3 mL), exposed for 1 h at 30 °C and then removed and dried. Significant CUR encapsulation rates were obtained, in the range 87–90%, as revealed by HPLC, which corresponded to a drug content in the range 3.2–6.9 mg (relative to 100 mg of hydrogel dry mass). The cumulative release of CUR in water was then studied over time, quantified by HPLC (Figure 6). **H2** and **H3** were immersed in deionized water and **H4** was immersed in phosphate buffer (pH 7.2–7.6) or sodium citrate buffer (pH 4.5–5.5) to assess degradation and release at the same time. The volume extracted at each time point was refilled with fresh water/buffer solution.

**Figure 6:**
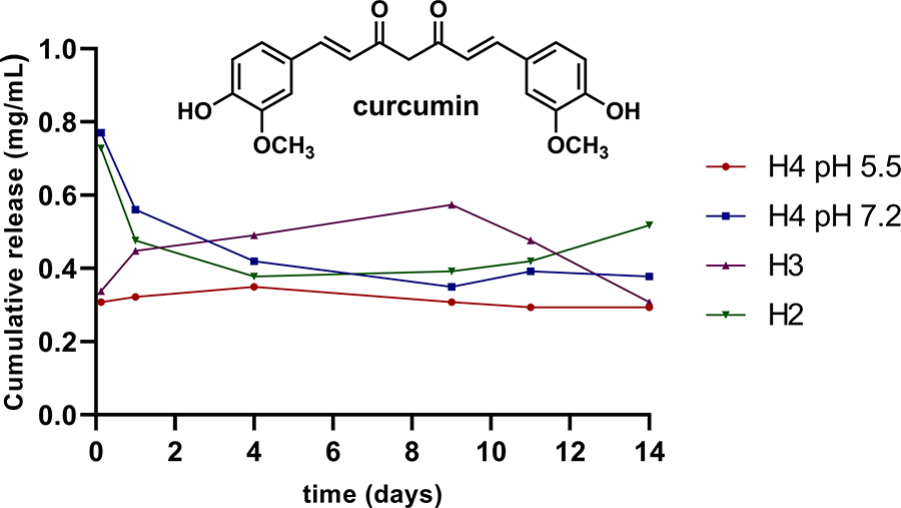
Cumulative release of curcumin of the different hydrogels evaluated **H2**, **H3** and **H4**, the latter in media at two different pH. Loading (per 100 mg hydrogel): 6.9 mg (**H2**), 3.2 mg (**H3**) and 3.6 mg (**H4**).

Although all hydrogels present very similar loading rates, the CUR release was different (Figure 6). PEG-based hydrogel **H2** showed maximum release when initially immersed in water solution, but then it progressively decreased. This indicated an internal nanostructuring of the hydrogel when immersed in water, which favors the reencapsulation of curcumin probably located on the external surface of the hydrogel. From day 4, a sustained release occurred that increased significantly far beyond the span of the experiment. This behavior resembled the one previously observed for carbosilane dendritic hydrogels bearing DTT or Pluronic L35 chains.^11,13^ The self-immolative hydrogel **H4** showed a similar pattern at pH 7.2, but without further release unlike **H2**. However, switching the pH to 5.5, clearly changed this pattern. The initial release was sustained from the time the hydrogel is immersed in the buffer, which can likely be attributed to **H4**’s degradation. Finally, **H3**, showed sustained release until day 9, when cumulative concentration began to decrease due to the dilution effect, as **H3** was presumably not degrading or only degrading very slowly. Overall, these results highlight the impact of the polymeric chains as well as the pH of the solution in the desired drug release.

## 4 Conclusions

The innovative bifunctional carbosilane dendrimers, comprising multiple alkyne peripheral groups and hydroxyl moieties in the core, are promising cross-linkers for the design of dendritic hydrogels with tunable degradation. The structural perfection, multivalent nature, lipophilicity, and stability offer unprecedented control over the synthesis of the networks as well as over the drug loading and release. Additionally, they enable the use of the highly efficient CuAAC click reaction under mild conditions, to provide cleavable and noncleavable hydrogels, and the hydroxyl pendant groups could be used to manipulate the mechanical properties of the hydrogels, as shown after reaction with glutaraldehyde. Overall, the dendritic cross-linkers clearly affected the network properties such as the cross-linking and swelling degrees, as well as their mechanical properties.

This study also showed that hydrogels degradation can be manipulated by carefully selecting the complementary polymer used, from very fast degradation using cleavable PEG **P1d** to a pH-tunable degradation using the self-immolative PEtG polymer **P3**, which increased at lower pH. The controlled degradation observed in the dendritic self-immolative hydrogel is highly desired in different applications, especially in the biomedical field. The polymer structure also affects drug release and enables sustained release over time, dependent on the pH of the solution. This pH-responsive behavior could be relevant in cancer applications, producing selective drug release in the tumor environment with lower pH. Overall, the carbosilane dendritic hydrogels reported herein represent a versatile and promising approach to improve the loading and controlled release of drugs with poor water solubility. They offer both synthetic precision and improved control over hydrogel degradation, thus opening new avenues in multiple biomedical applications.

## Supplementary Material



## Notes

Structural characterization of dendritic compounds (^1^H NMR, ^13^C NMR, ^1^H–^13^C-HSQC, MALDI-TOF spectra) and polymers (FTIR, SEC spectra); and degradation study of the self-immolative hydrogel (PDF)
