# Supplementary material for: Functional Self-Immolative Hydrogels with Dendritic Cross-Linkers for Controlled Drug Delivery
Source: Chem Mater. 2025 Jul 18;37(15):5814–24. doi: 10.1021/acs.chemmater.5c01006 (PMC12355646; doi:10.1021/acs.chemmater.5c01006)
Supplement: Supplementary file 1 [file cm5c01006_si_001.pdf]

Functional Self-Immolative Hydrogels with Dendritic Crosslinkers for Controlled Drug Delivery

Silvia Muñoz-Sánchez,[a] Jue Gong,[b] Francisco Javier de la Mata,[a, c, d] Elizabeth R. Gillies,[b,e] and Sandra García-Gallego [a,c,d]*

a. University of Alcalá, Department of Organic and Inorganic Chemistry and Research Institute in Chemistry "Andrés M. Del Río" (IQAR), 28805, Madrid, Spain. silvia.munoz@uah.es

b. Department of Chemistry, The University of Western Ontario, London, Ontario, N6A 5B7 Canada.

c. Networking Research Center on Bioengineering, Biomaterials and Nanomedicine (CIBER-BBN), 28029, Madrid, Spain;

d. Institute Ramón y Cajal for Health Research (IRYCIS), 28034, Madrid, Spain.

e. Department of Chemical and Biochemical Engineering, The University of Western Ontario, London, Ontario, N6A 5B9 Canada

KEYWORDS. Hydrogel; dendrimer; self-immolative; polymer; drug delivery.

Content

Figure S1. ^{1}H and ^{13}C NMR spectra of dendron BrG2E$_2$ **(1)** in CDCl$_3$. 5

Figure S2. ^{1}H-^{13}C HSQC spectra of dendron BrG2E$_2$ **(1)** in CDCl$_3$. 5

Figure S3. Mass spectrum of dendrimer BrG2E$_4$ **(1)**. 6

Figure S4. ^{1}H and ^{13}C NMR spectra of dendrimer BrG3E$_4$ **(2)** in CDCl$_3$. 6

Figure S5. ^{1}H-^{13}C HSQC spectra of dendrimer BrG3E$_4$ **(2)** in CDCl$_3$. 7

Figure S6. Mass spectrum of dendrimer BrG3E$_4$ **(2)**. 7

Figure S7. ^{1}H-NMR spectra of dendron BrG4E$_8$ **(3)**. 8

Figure S8. ^{1}H and ^{13}C NMR spectra of dendrimer N$_2$O$_2$-G2E$_4$ **(4)** in CDCl$_3$. 8

Figure S9. ^{1}H-^{13}C HSQC spectra of dendrimer N$_2$O$_2$-G2E$_4$ **(4)** in CDCl$_3$. 9

Figure S10. MALDI spectrum of dendrimer N$_2$O$_2$-G2E$_4$ **(4)**. 9

Figure S11. ^{1}H and ^{13}C NMR spectra of dendrimer N$_2$O$_2$-G3E$_8$ **(5)** in CDCl$_3$. 10

Figure S12. ^{1}H-^{13}C HSQC spectra of dendrimer N$_2$O$_2$-G3E$_8$ **(5)** in CDCl$_3$. 10

Figure S13. MALDI spectra of dendrimer N$_2$O$_2$-G3E$_8$ **(5)**. 10

Figure S14. ^{1}H and ^{13}C NMR spectra of PEG(N$_3$)$_2$ **(6)** in CDCl$_3$. 11

Figure S15. ^{1}H-^{13}C HSQC spectra of PEG(N$_3$)$_2$ **(6)** in CDCl$_3$. 11

Figure S16. ^{1}H and ^{13}C NMR spectra of PEGCOO(N$_3$)$_2$ **(7)** in CDCl$_3$. 12

Figure S17. ^{1}H-^{13}C HSQC spectra of PEGCOO(N$_3$)$_2$ **(7)** in CDCl$_3$. 12

Figure S18. ^{1}H-NMR spectra of PEtG-M **(8)** in CDCl$_3$. 13

Figure S19. ^{13}C-NMR spectra of PEtG-M **(8)** in CDCl$_3$. 13

Figure S20. ^{1}H-NMR spectra of PEtG-EVE **(9)** in CDCl$_3$. 14

Figure S21. ^{13}C-NMR spectra of PEtG-EVE **(9)** in CDCl$_3$. 14

Figure S22. ^{1}H-NMR spectra of TEG-N$_3$-30-PGAm-M **(10)** in CDCl$_3$. 15

Figure S23. ^{13}C-NMR spectra of TEG-N$_3$-30-PGAm-M **(10)** in CDCl$_3$. 15

Figure S24. ^{1}H-NMR spectra of TEG-N$_3$-30-PGAm-EVE **(11)** in CDCl$_3$. 16

Figure S25. ^{13}C-NMR spectra of TEG-N$_3$-30-PGAm-EVE **(11)** in CDCl$_3$. 16

Figure S26. FT-IR spectra of PEtG-M, PEtG-EVE, TEG-N$_3$-30-PGAm-M and TEG-N$_3$-30-PGAm-EVE. 17

Figure S27. Size exclusion chromatograms of PEtG-M, PEtG-EVE, TEG-N3-30-PGAm-M and TEG-N3-30-PGAm-EVE 17

Figure S28. FT-IR spectrum of hydrogel H2 (top), compared to the precursor de 18

Figure S29. FT-IR spectrum of hydrogel H4, ... 18

Figure S30. Degradation study of the self-immolative hydrogel H4 at pH 7.2, as monitored through 1H-NMR in D2O, ... 19

Figure S31. Degradation study of the self-immolative hydrogel **H4** at pH 5.5, as monitored through rheology (change in G'). 19

Bibliography 20

Materials and methods

Materials. Reagents and solvents were purchased from commercial sources and used as received unless otherwise noted. 2-Azidoethylamine[1] and Tri(ethylene glycol)methyl ether amine (TEG-amine)[2] were synthesized as previously reported. Alkyne-functionalized *o*-nitrobenzyl alcohol was synthesized as previously reported. EtG in toluene solution (50% w/w) were obtained from Alfa Aesar (Ward Hill, MA, USA). Ethyl glyoxylate (EtG) was purified by distillation over P_2O_5 as previously reported.[3] 1-Butanol, dichloromethane (DCM), methanol and trifluoroacetic acid (TFA) were purchased from Caledon Laboratory Chemicals (Georgetown, ON, Canada). Toluene and ethyl acetate were purchased from Thermo Fisher Scientific (Burlington, ON, Canada). The following reagents were purchased from Sigma-Aldrich (Oakville, ON, Canada): ethyl vinyl ether, triethylamine (NEt_3), 1,4-dioxane, dimethyl sulfoxide-d_6 (99.9 atom% D), chloroform-*d* (99.8 atom% D) and deuterium oxide (99.9 atom% D). CH_2Cl_2 and NEt_3 were distilled over CaH_2 under nitrogen at atmospheric pressure before use. Toluene was distilled over sodium with benzophenone as the indicator under nitrogen at atmospheric pressure before use. 1-Butanol was dried over 4A molecular sieves and distilled under nitrogen at atmospheric pressure before use. Toluene was distilled over Na/benzophenone and NEt_3 was distilled over CaH_2. Dry dioxane was obtained from Sigma-Aldrich.

General methods. ^{1}H and ^{13}C NMR spectra were obtained using a 400 MHz Bruker AvIII HD and Bruker AVANCE Neo 400 instruments. ^{1}H NMR chemical shifts are reported in ppm and are referenced to the residual solvent signals of $CDCl_3$ (7.26 ppm), D_2O (4.79 ppm) or DMSO-d_6 (2.50 ppm) while ^{13}C NMR chemical shifts were referenced to the residual solvent signals of $CDCl_3$ (77.2 ppm). HSQC experiments were also carried out under these conditions. FT-IR spectra were obtained using a PerkinElmer FT-IR Spectrum Two instrument in attenuated total reflectance mode. SECs in THF were obtained using an instrument equipped with a Viscotek GPC Max VE2001 solvent module, a Viscotek VE3580 RI detector, and two Agilent PolyPore (300 × 7.5mm) columns connected in series to a PolyPore guard column. Samples were dissolved in THF (glass-distilled grade) at a concentration of ~5 mg/mL, filtered through a 0.2 μm polytetrafluoroethylene (PTFE)

syringe filter, and injected using a 100 μL loop. Samples were run at a flow rate of 1 mL/min for 30 min at 30◦C. Number average molar mass (M_n) and dispersity ($Đ$) were determined relative to PEG standards. SECs in DMF were obtained using an instrument equipped with a Waters 515 HPLC pump, Waters In-Line Degasser AF, two PLgel mixed D 5 μm (300 ×1.5 mm) columns attached to a corresponding PLgel guard column, and a Wyatt Optilab Rex RI detector. Samples were dissolved in DMF containing 10 mM LiBr and 1% (v/v) NEt_3 at a concentration of ~5 mg/mL and filtered through a 0.2 μm PTFE syringe filter prior to injection using a 50 μL loop. Samples were run at a flow rate of 1 mL/min for 30 min at 85◦C. M_n and $Đ$ values were determined relative to poly(methyl methacrylate) (PMMA) standards. The viscoelastic properties of hydrogels were measured using a Discovery Hybrid Rheometer 10 (DHR-10) from TA Instruments (New Castle, DE, USA) at 25◦C using parallel-plate geometry (8 mm diameter). Amplitude sweep (0.1–100% strain at 1 Hz) and frequency sweep (0.1–10 Hz at 1% strain) measurements were performed.

Swelling studies. The swelling assays were performed by immersing the hydrogels in a distilled water bath for two days. The swelling degree (SD) was calculated with the following equation: $SD\% = ((W_S - W_D) / W_D) * 100$

where W_S is the weight of the swollen gel at the different time points and W_D is the weight of the dry gel after purification. All gels were tested in duplicate.

Crosslinking studies. The gel fraction (GF) was calculated using the following equation:

$GF(\%) = (W_D / W_C) * 100$

where W_C is the overall weight of the dry mass after click reaction and W_D is the weight of the dry mass after purification. All measures were performed in duplicate.

High Performance Liquid Chromatography (HPLC). Curcumin release studies were performed at CAIQ-UAH on Agilent 1200 HPLC equipment, using an ACE Excel 5 column and a mobile phase of 0.1% aqueous H_3PO_4 and acetonitrile (30:70), with an injection volume of 10 μL. The drug was detected at a wavelength of 425 nm.

Figures

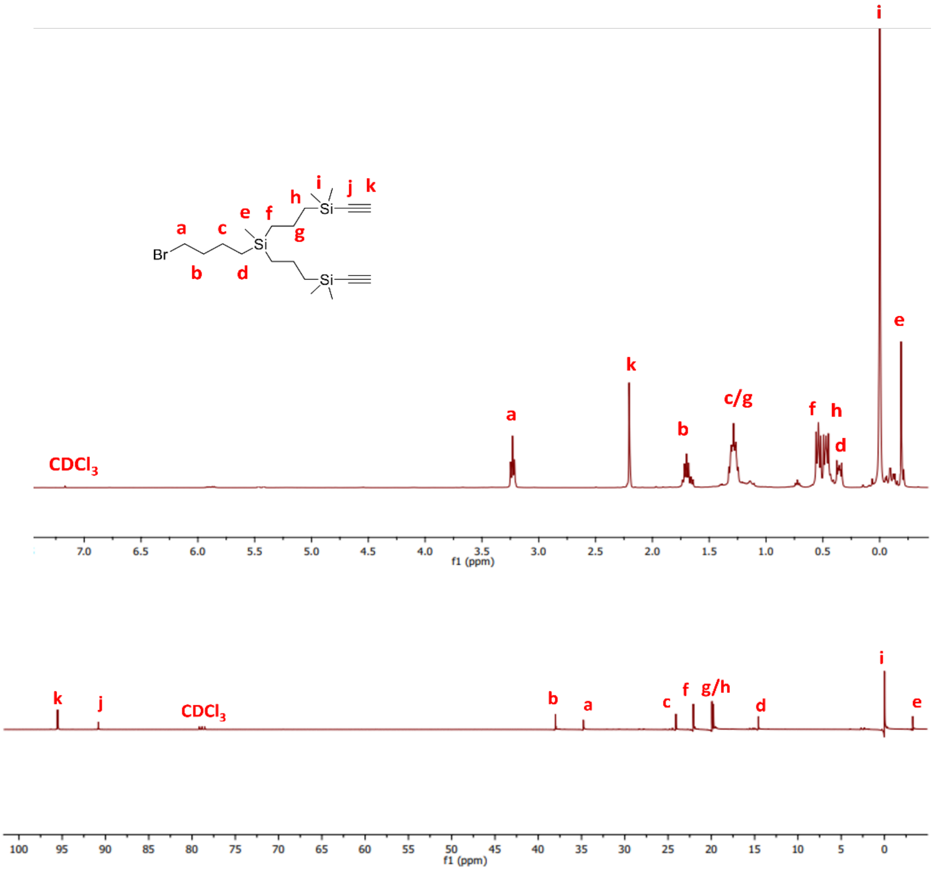

Figure S1. ^{1}H and ^{13}C NMR spectra of dendron BrG2E$_2$ **(1)** in $CDCl_3$.

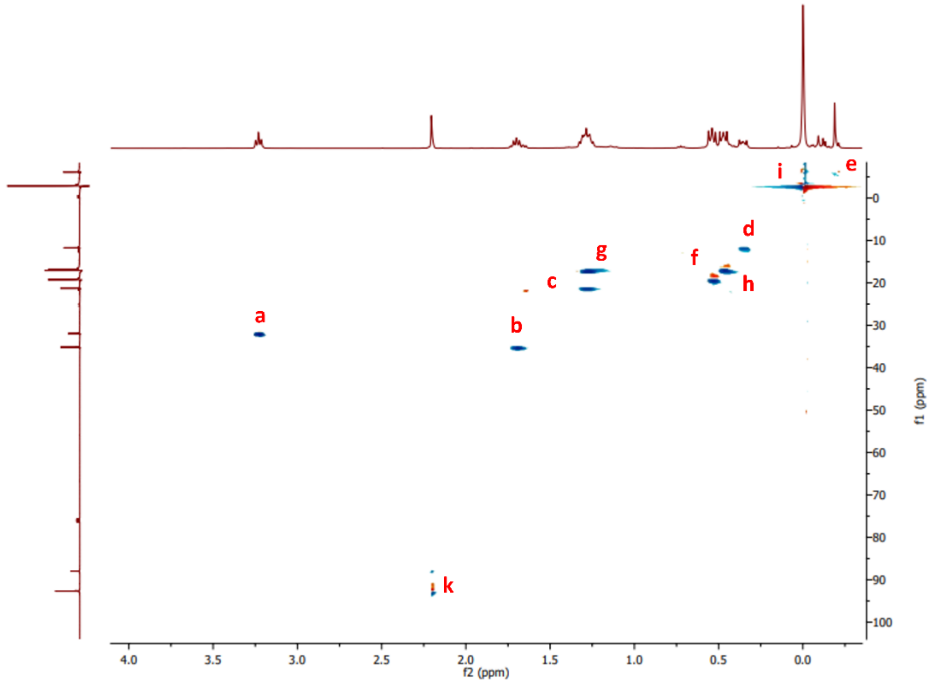

Figure S2. ^{1}H-^{13}C HSQC spectra of dendron BrG2E$_2$ **(1)** in $CDCl_3$.

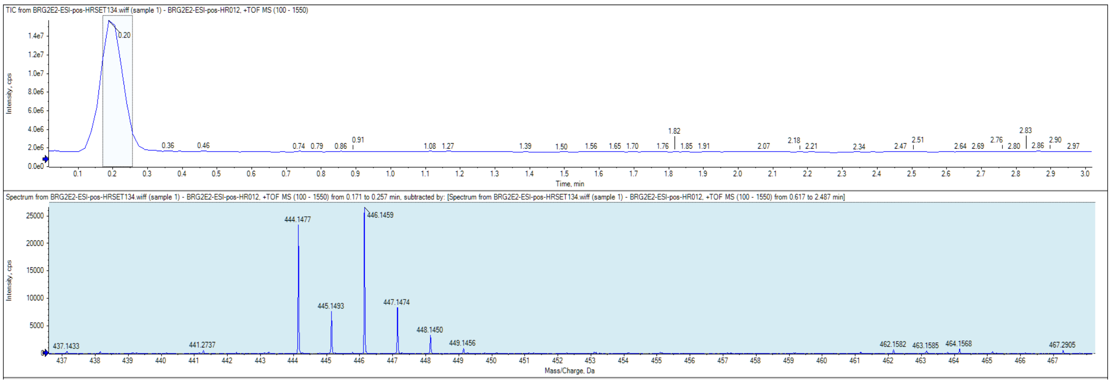

Figure S3. Mass spectrum of dendrimer BrG2E$_4$ **(1)**.

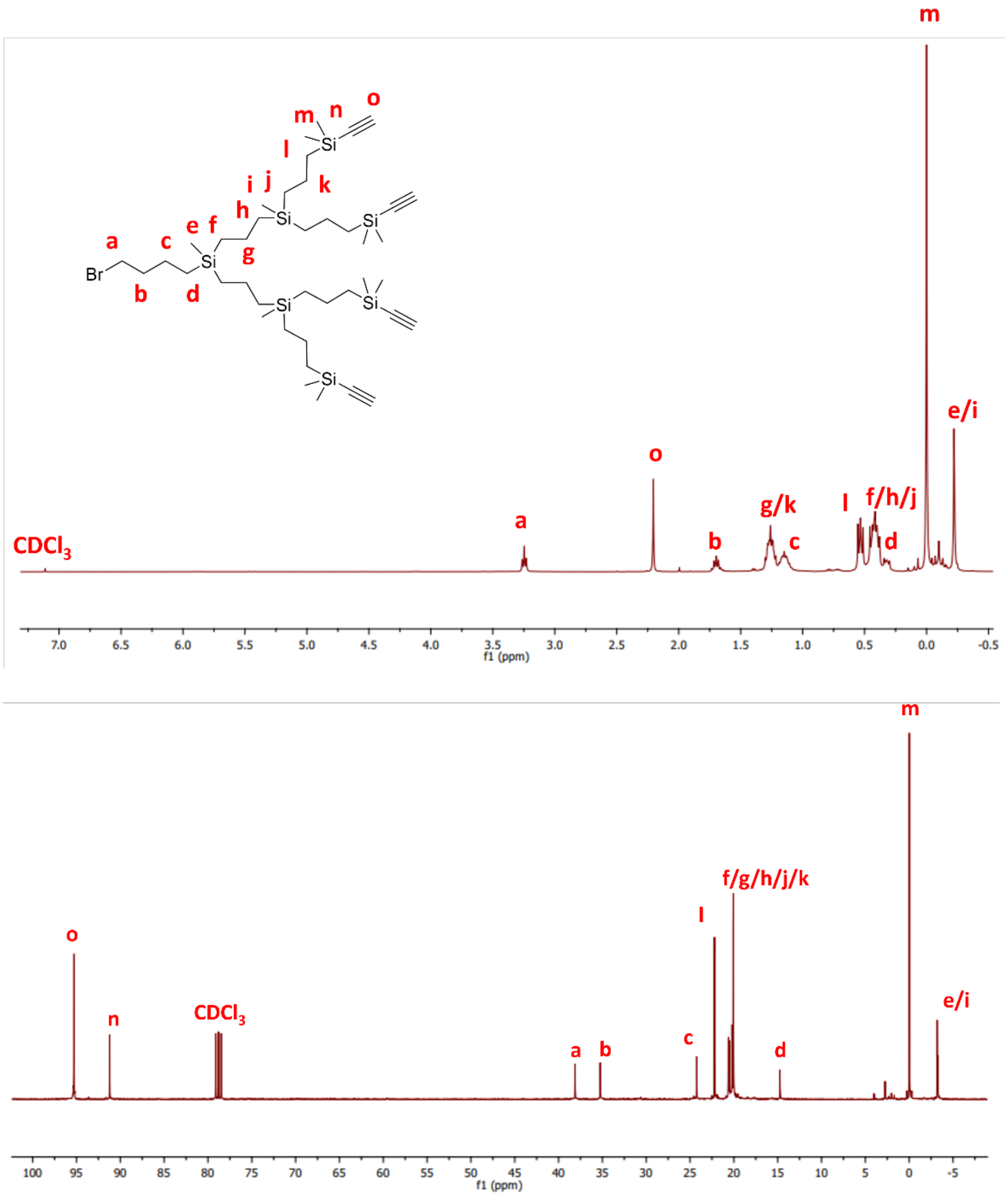

Figure S4. ^{1}H and ^{13}C NMR spectra of dendrimer BrG3E$_4$ **(2)** in CDCl$_3$.

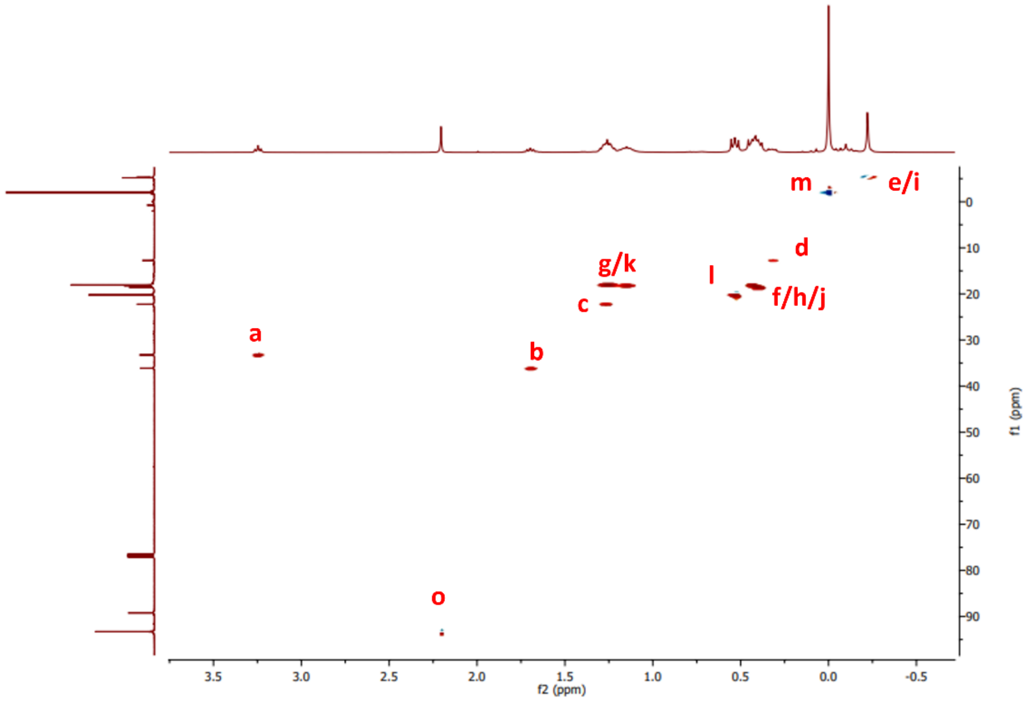

Figure S5. ^1H-^{13}C HSQC spectra of dendrimer BrG3E$_4$ **(2)** in $CDCl_3$.

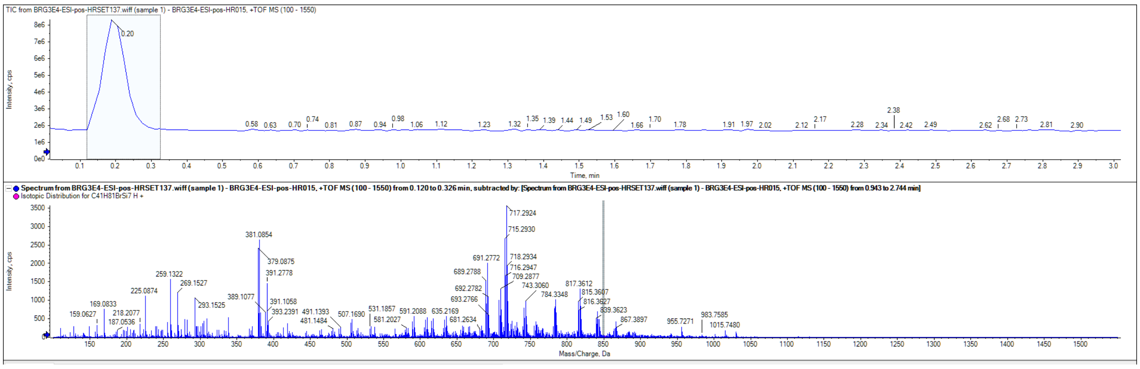

Figure S6. Mass spectrum of dendrimer BrG3E$_4$ **(2)**.

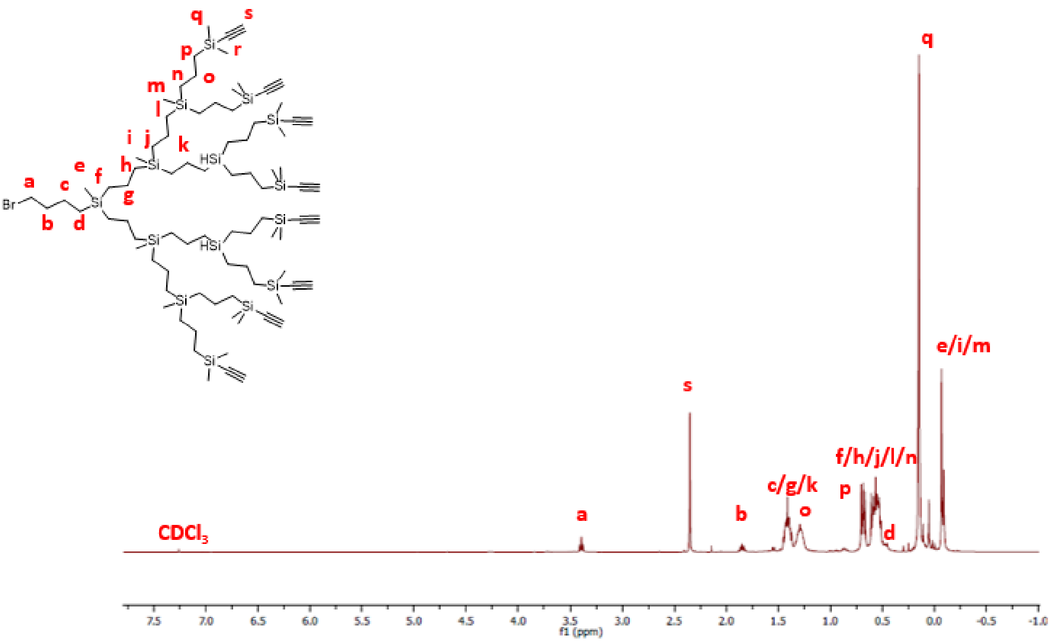

Figure S7. ^{1}H-NMR spectra of dendron BrG4E$_8$ **(3)**.

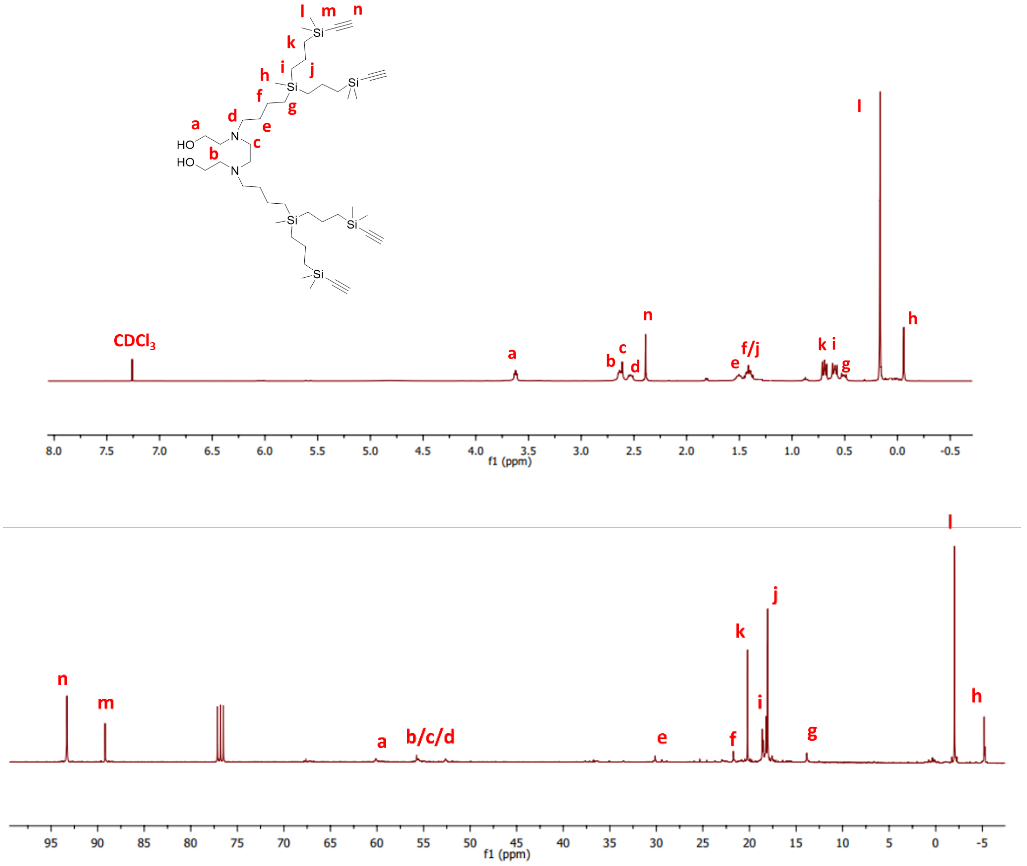

Figure S8. ^{1}H and ^{13}C NMR spectra of dendrimer N_2O_2-G2E$_4$ **(4)** in $CDCl_3$.

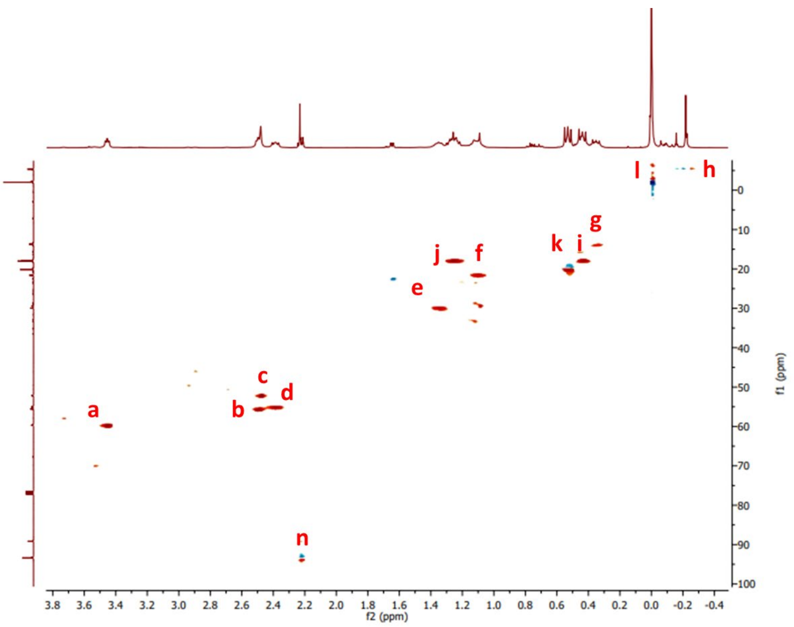

Figure S9. ^{1}H-^{13}C HSQC spectra of dendrimer N_2O_2-G2E$_4$ **(4)** in $CDCl_3$.

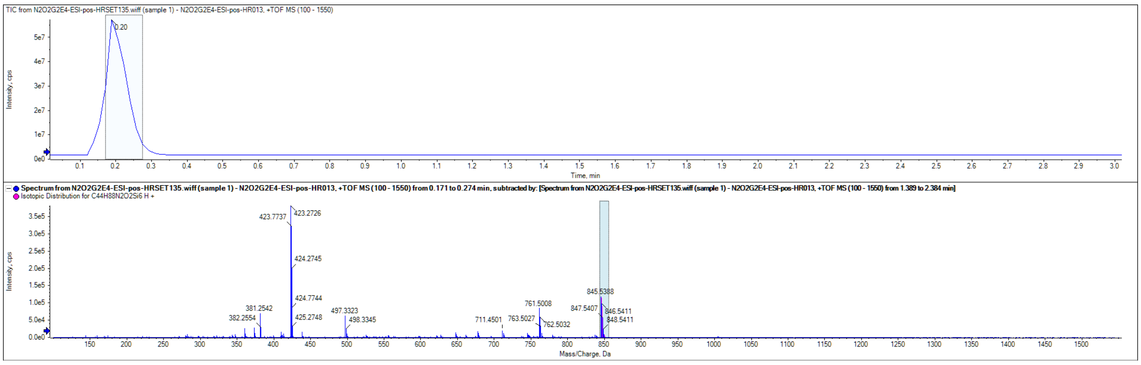

Figure S10. MALDI spectrum of dendrimer N_2O_2-G2E$_4$ **(4)**.

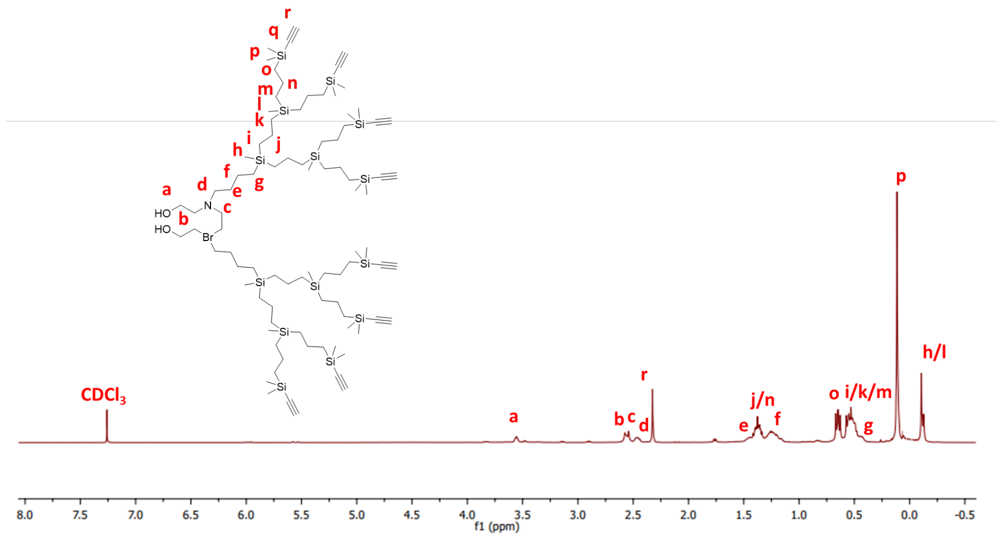

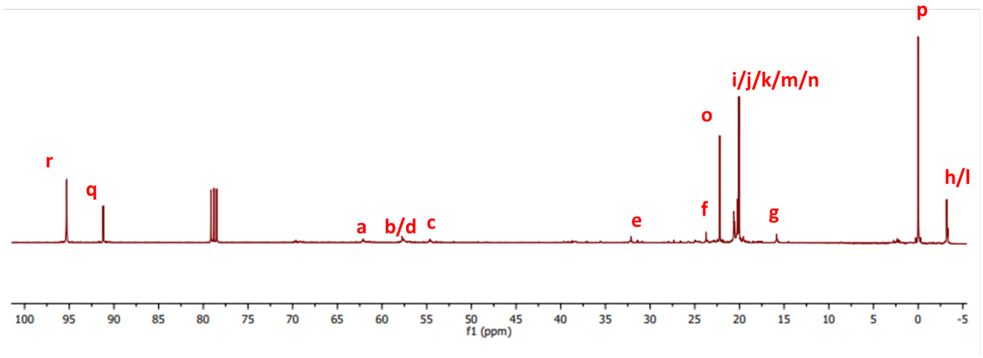

Figure S11. ^{1}H and ^{13}C NMR spectra of dendrimer N_2O_2-G3E$_8$ **(5)** in $CDCl_3$.

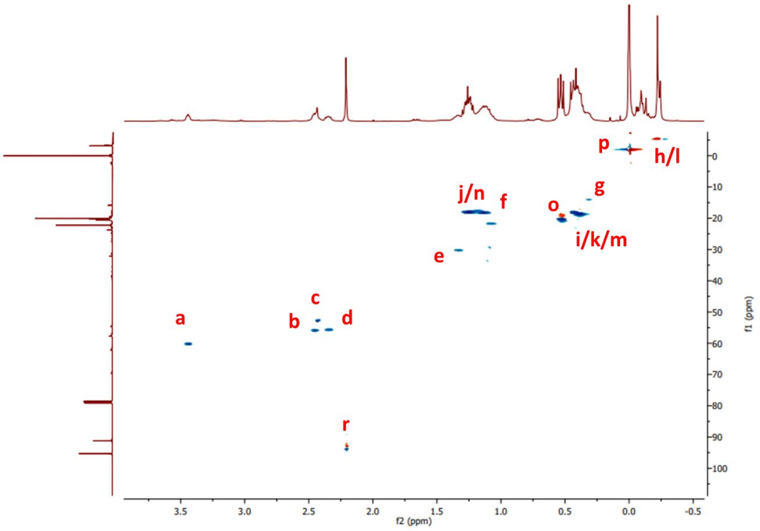

Figure S12. ^{1}H-^{13}C HSQC spectra of dendrimer N_2O_2-G3E$_8$ **(5)** in $CDCl_3$.

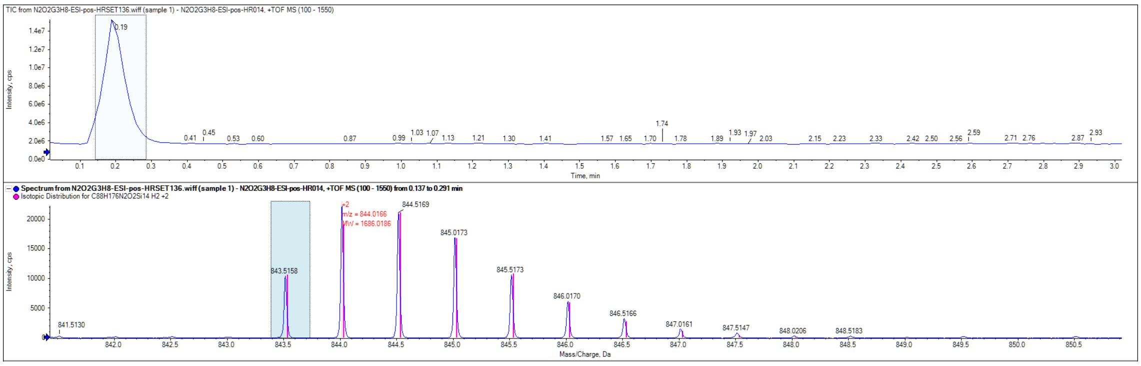

Figure S13. MALDI spectra of dendrimer N_2O_2-G3E$_8$ **(5)**.

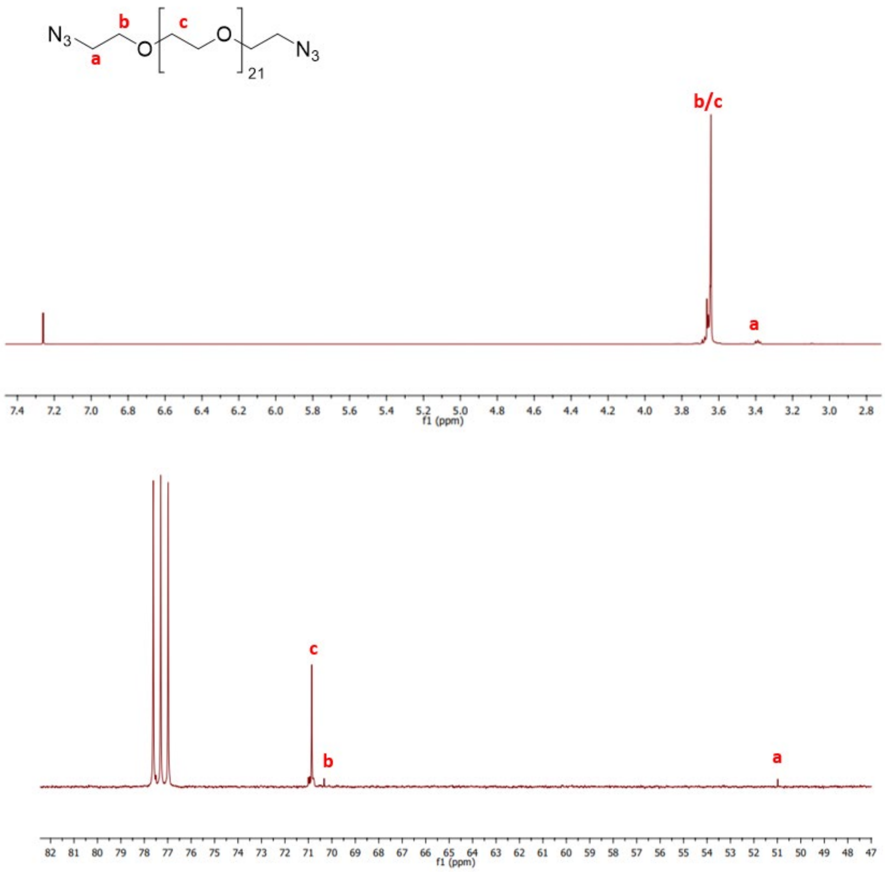

Figure S14. ^{1}H and ^{13}C NMR spectra of PEG$(N_3)_2$ **(6)** in $CDCl_3$.

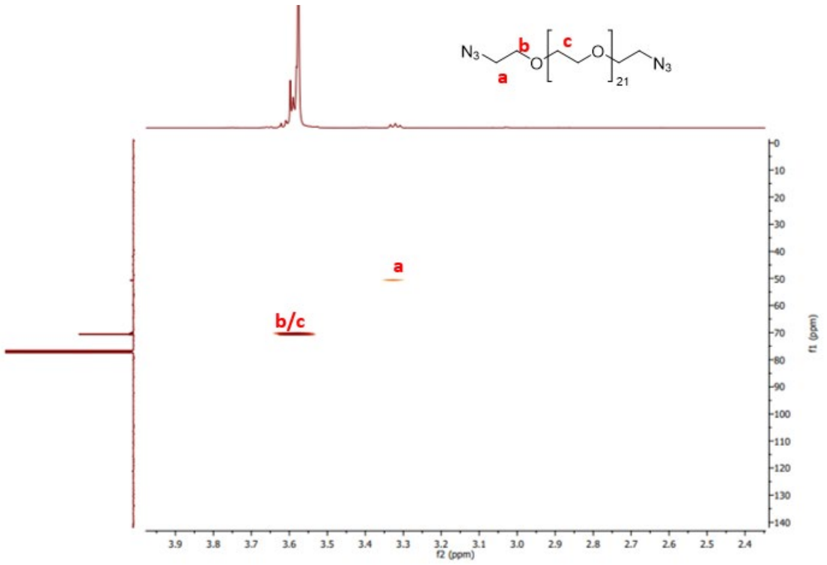

Figure S15. ^{1}H-^{13}C HSQC spectra of PEG$(N_3)_2$ **(6)** in $CDCl_3$.

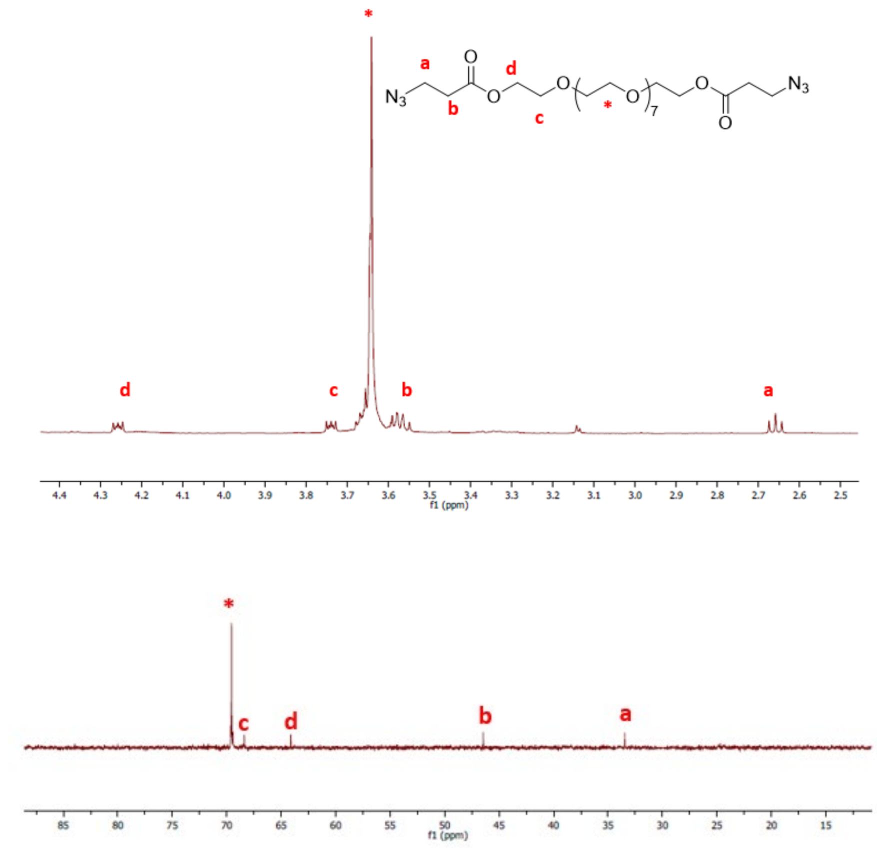

Figure S16. ^{1}H and ^{13}C NMR spectra of PEGCOO(N_3)$_2$ **(7)** in $CDCl_3$.

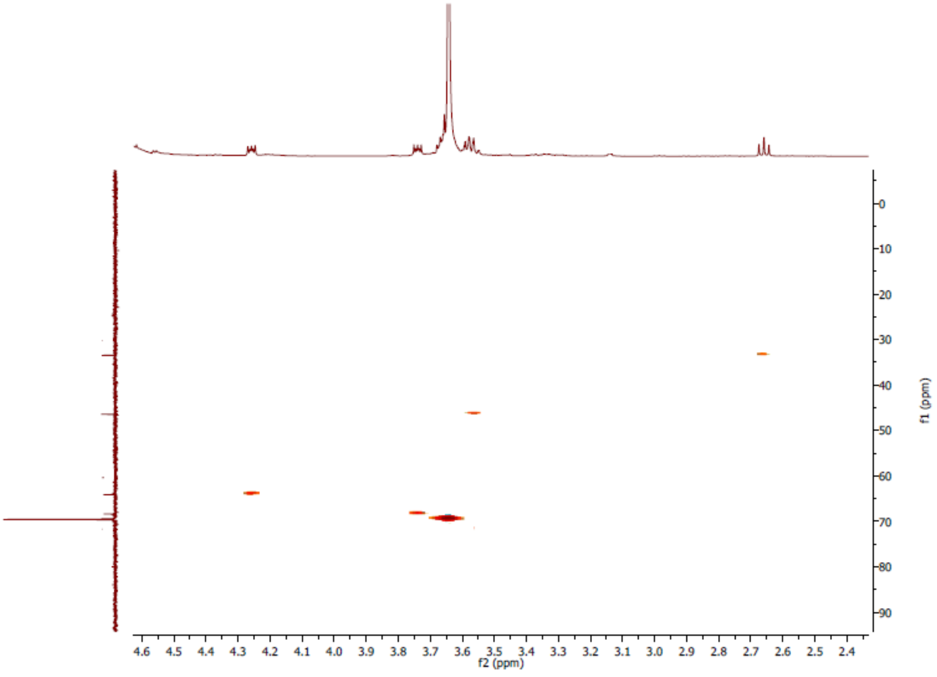

Figure S17. ^{1}H-^{13}C HSQC spectra of PEGCOO(N_3)$_2$ **(7)** in $CDCl_3$.

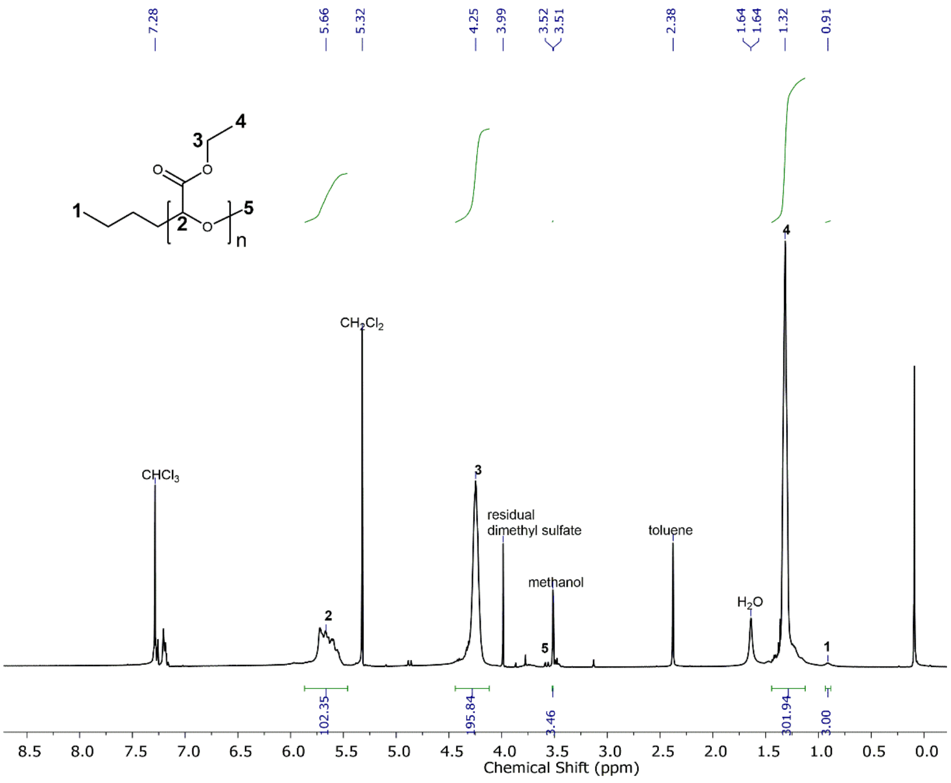

Figure S18. ^{1}H-NMR spectra of PEtG-M **(8)** in $CDCl_3$.

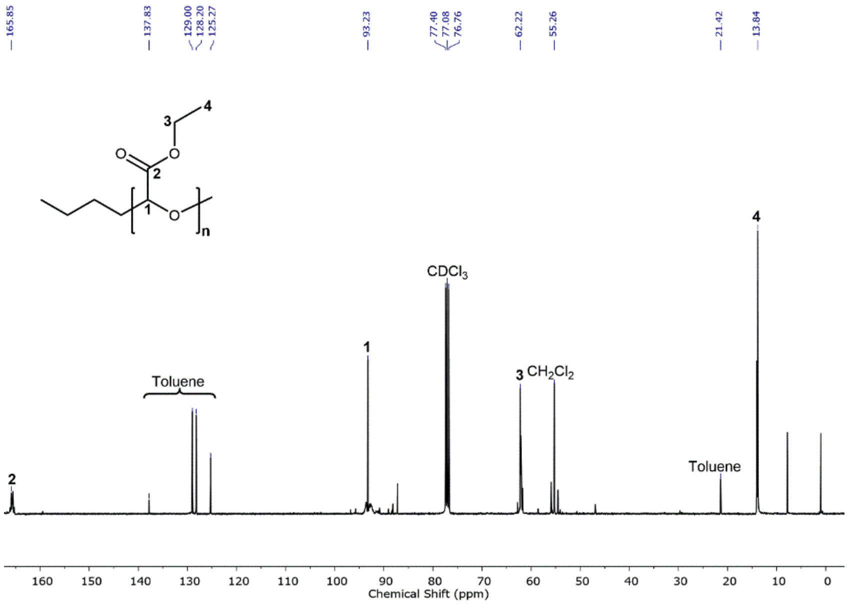

Figure S19. ^{13}C-NMR spectra of PEtG-M **(8)** in $CDCl_3$.

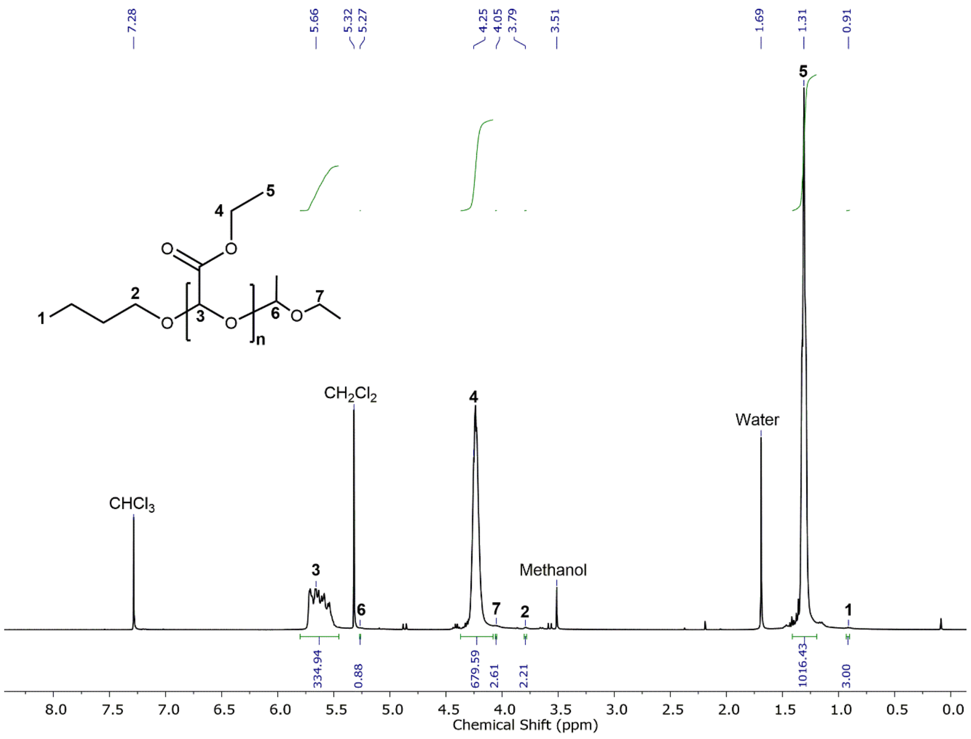

Figure S20. ^{1}H-NMR spectra of PEtG-EVE **(9)** in $CDCl_3$.

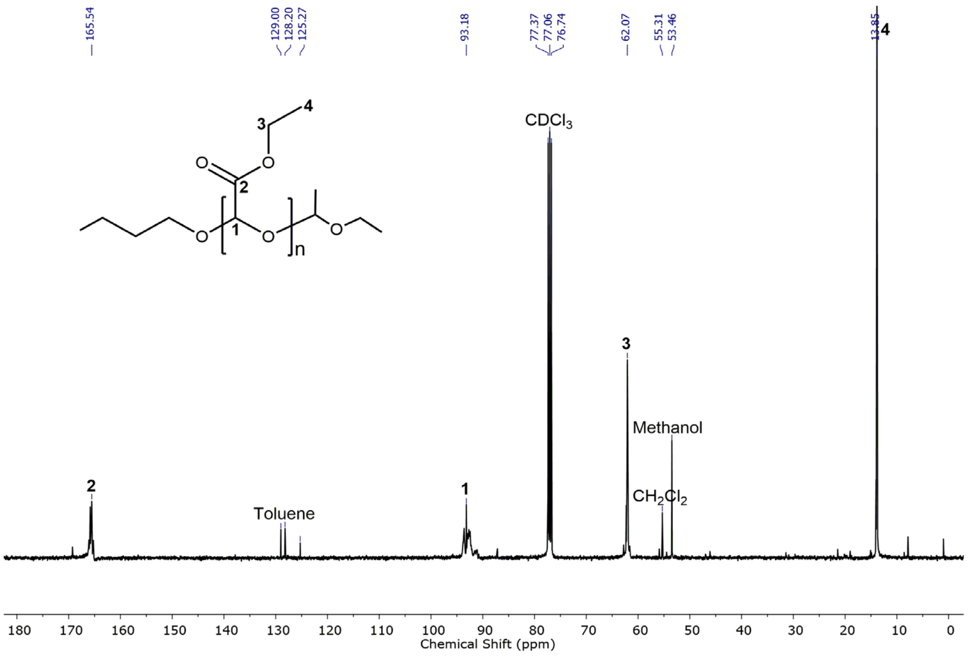

Figure S21. ^{13}C-NMR spectra of PEtG-EVE **(9)** in $CDCl_3$.

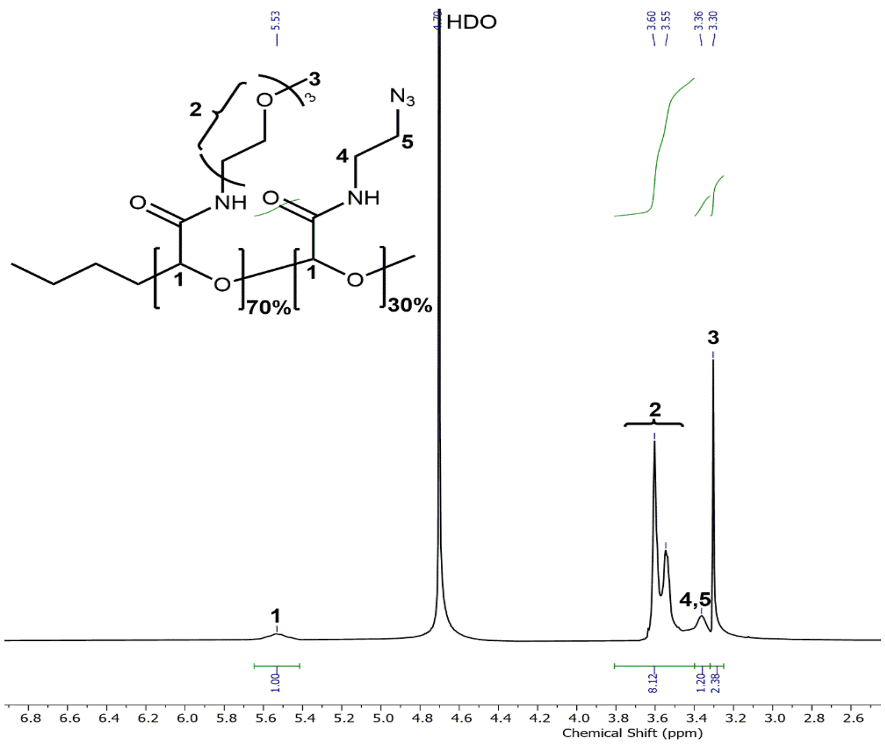

Figure S22. ^{1}H-NMR spectra of TEG-N_3-30-PGAm-M **(10)** in $CDCl_3$.

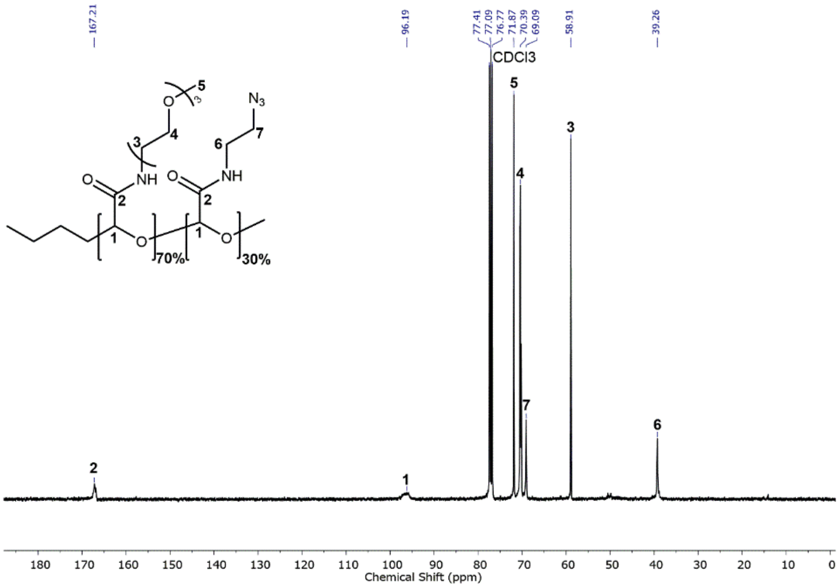

Figure S23. ^{13}C-NMR spectra of TEG-N_3-30-PGAm-M **(10)** in $CDCl_3$.

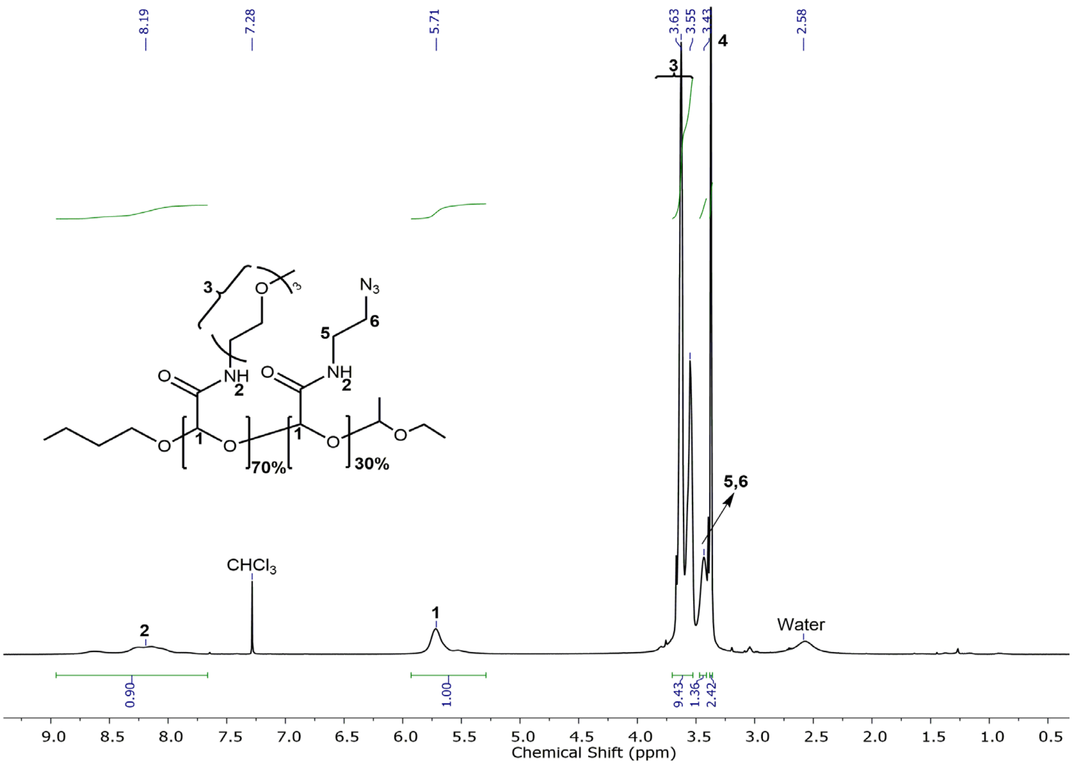

Figure S24. ^{1}H-NMR spectra of TEG-N_3-30-PGAm-EVE **(11)** in $CDCl_3$.

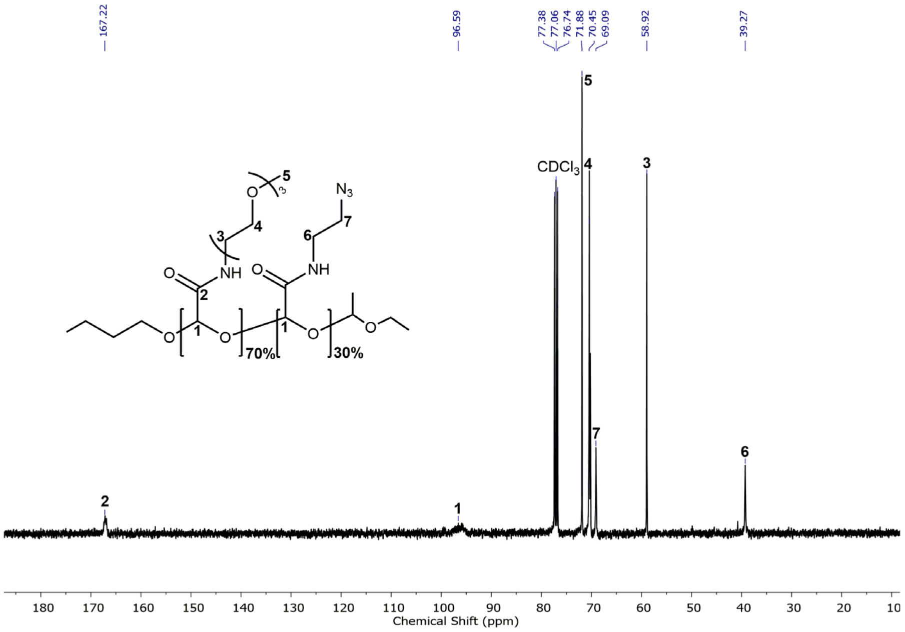

Figure S25. ^{13}C-NMR spectra of TEG-N_3-30-PGAm-EVE **(11)** in $CDCl_3$.

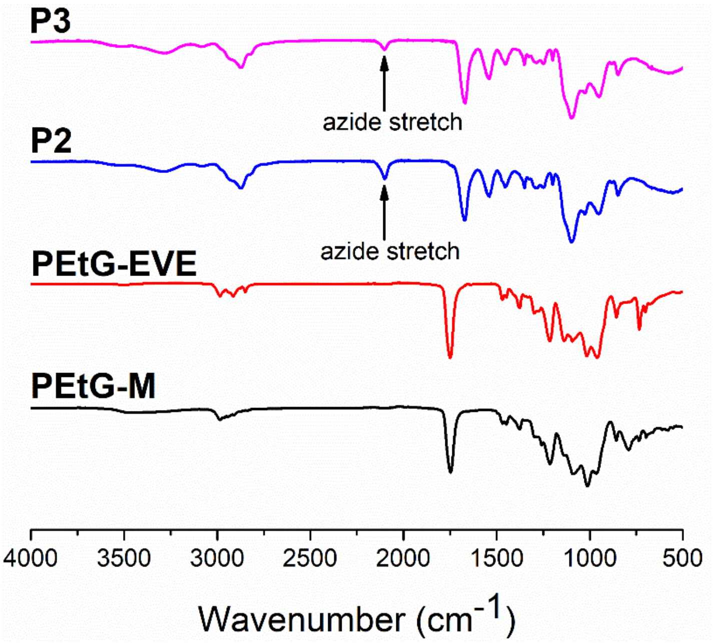

Figure S26. FT-IR spectra of PEtG-M, PEtG-EVE, TEG-N_3-30-PGAm-M and TEG-N_3-30-PGAm-EVE

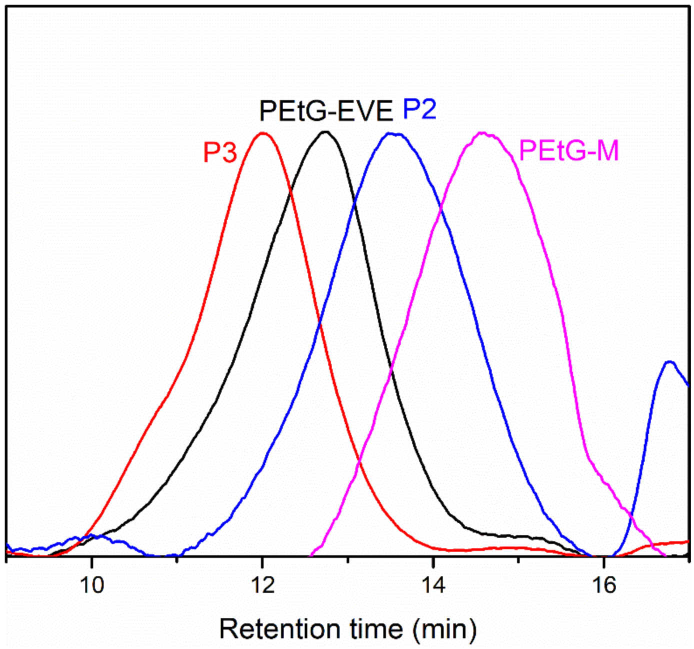

Figure S27. Size exclusion chromatograms of PEtG-M, PEtG-EVE, TEG-N3-30-PGAm-M and TEG-N3-30-PGAm-EVE (in DMF with refractive index (RI) detection).

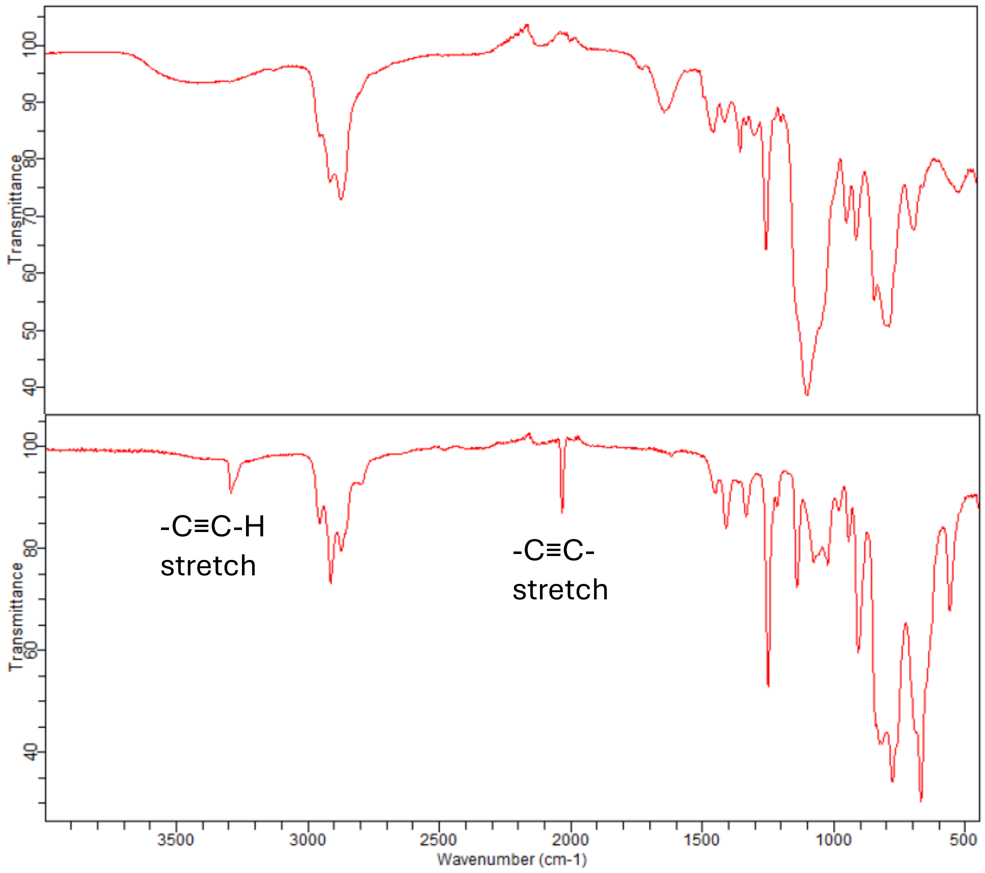

Figure S28. FT-IR spectrum of hydrogel H2 (top), compared to the precursor dendrimer N2O2G3E$_8$ (bottom).

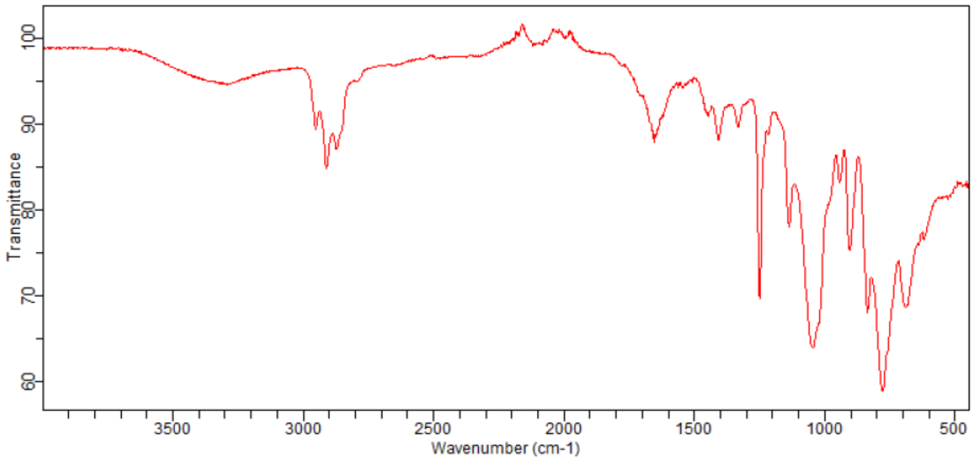

Figure S29. FT-IR spectrum of hydrogel H4, formed from N2O2G3E$_8$ and PGAm-EVE.

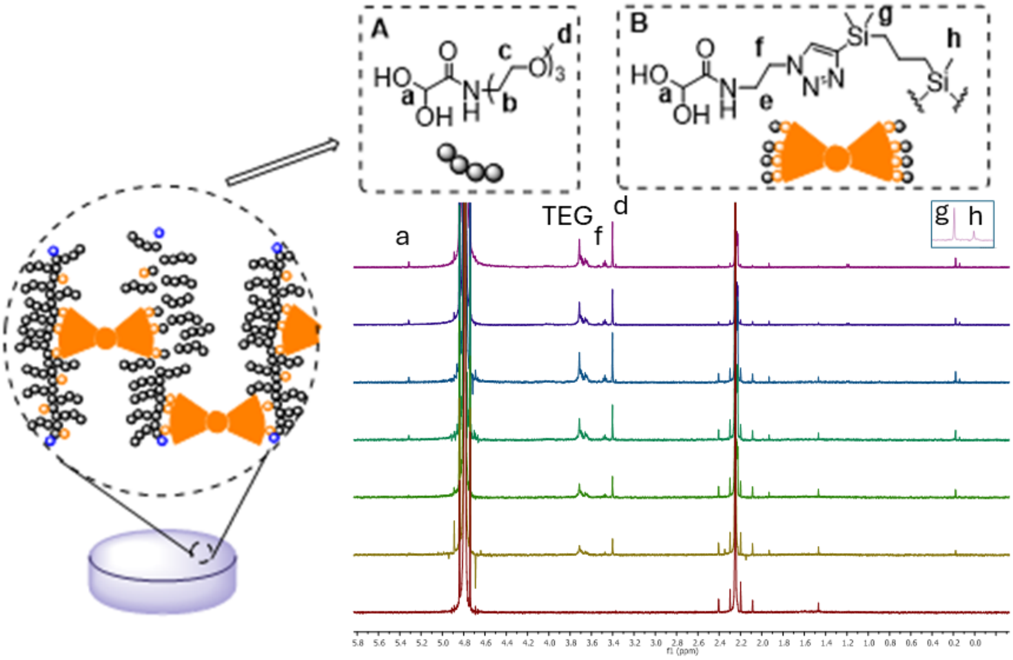

Figure S30. Degradation study of the self-immolative hydrogel H4 at pH 7.2, as monitored through 1H-NMR in D2O, highlighting the signals of released compounds A and B over time (from bottom to top: degradation products at 3, 24, 48, 72 h, then 7, 14 and 21 days.

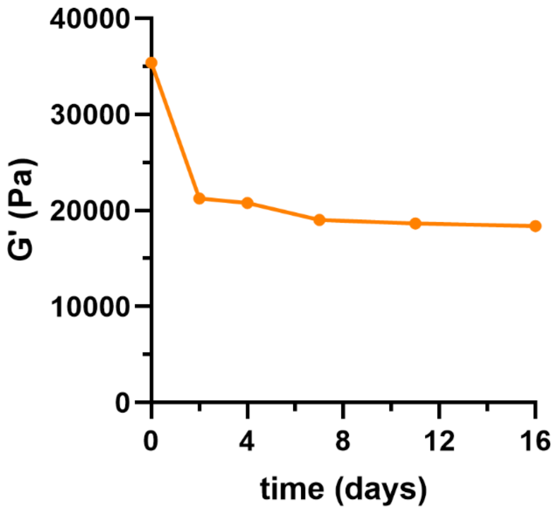

Figure S31. Degradation study of the self-immolative hydrogel **H4** at pH 5.5, as monitored through rheology (change in G').

Bibliography

1. Hannant, J.; Hedley, J. H.; Pate, J.; Walli, A.; Farha Al-Said, S. A.; Galindo, M. A.; Connolly, B. A.; Horrocks, B. R.; Houlton, A.; Pike, A. R. Modification of DNA-Templated Conductive Polymer Nanowires via Click Chemistry. *Chem. Commun.* **2010**, *46*, 5870-5872.
2. Barnes, B. E.; Jenkins, T. A.; Stein, L. M.; Mathers, R. T.; Wicaksana, M.; Pasquinelli, M. A.; Savin, D. A. Synthesis and Characterization of a Leucine-Based Block Co-Polypeptide: The Effect of the Leucine Zipper on Self-Assembly. *Biomacromolecules* **2020,** *21*, 2463-2472.
3. Kenaree, A. R.; Gillies, E. R. Controlled Polymerization of Ethyl Glyoxylate Using Alkyllithium and Alkoxide Initiators. *Macromolecules* **2018**, *51*, 5501-5510
